# Impaired emotional memory dissipation in insomnia disorder

**DOI:** 10.1017/S0033291725101566

**Published:** 2025-09-01

**Authors:** Shengzi Zeng, Hao Fong Sit, Xiao Li, Ryan Bottary, Edward F. Pace-Schott, Tony J. Cunningham, Shirley Xin Li, Xiaoqing Hu

**Affiliations:** 1 Department of Psychology, The University of Hong Kong, Hong Kong SAR, China; 2 Center for Sleep and Cognition, Department of Psychiatry, Beth Israel Deaconess Medical Center, Boston, MA, USA; 3 Division of Sleep Medicine, Harvard Medical School, Boston, MA, USA; 4 Institute for Graduate Clinical Psychology, Widener University, Chester, PA, USA; 5 Department of Psychiatry, Mass General Brigham, Charlestown, MA, USA; 6 Athinoula A. Martinos Center for Biomedical Imaging, Charlestown, MA, USA; 7 The State Key Laboratory of Brain and Cognitive Sciences, The University of Hong Kong, Hong Kong SAR, China; 8 HKU-Shenzhen Institute of Research and Innovation, Shenzhen, China

**Keywords:** sleep, emotional memory, insomnia disorder, EEG, multivariate pattern analysis

## Abstract

**Background:**

Insomnia disorder, characterized by chronic sleep disruption, often co-occurs with maladaptive emotional memory processing. However, much remains unknown regarding the evolution of emotional memories and their neural representations over time among individuals with insomnia disorder.

**Method:**

We examined the electroencephalographic (EEG) activities during emotional memory encoding, post-encoding sleep, and multiple retrieval phases – including immediate post-encoding, post-sleep, and a 7-day delayed retrieval – among 34 participants with insomnia disorder and 35 healthy control participants.

**Results:**

Healthy controls exhibited adaptive dissipation of emotional memory: memory declined over time, accompanied by reduced subjective feelings toward negative memories. In contrast, participants with insomnia exhibited impaired dissipation: they retained both the emotional content and affective tone of the memories, with diminished time-dependent declines in memory and affect. Beyond behavioral performance, only participants with insomnia maintained stable neural representations of emotion over time, a pattern absent in healthy controls. Additionally, during the post-encoding sleep, slow-wave sleep (SWS), and rapid eye movement (REM) sleep durations predicted the adaptive dissipation of emotional memory over time, but only among healthy participants.

**Conclusion:**

These findings highlight abnormalities in emotional memory processing among individuals with insomnia disorder and underscore the important function of SWS and REM sleep in facilitating adaptive emotional memory processing.

## Introduction

Insomnia disorder, characterized by persistent difficulties in sleep initiation, maintenance, or early awakening, has emerged as a significant public health concern due to its high prevalence (Adjaye-Gbewonyo, Ng, & Black, 2022; American Psychiatric Association, 2013; Wittchen et al., 2011). Beyond nighttime disruption, insomnia is also associated with impaired daytime cognitive functioning and altered emotional processing, which in turn increase vulnerability to psychiatric disorders including depression, anxiety, and posttraumatic stress disorder (PTSD) (Cerolini, 2015; Hertenstein, Benz, Schneider, & Baglioni, 2023; Palagini, Bastien, Marazziti, Ellis, & Riemann, 2019). Comorbidity between insomnia and mood-related disorders is especially high (Tsuno, Besset, & Ritchie, 2005). For example, about two thirds of individuals with major depressive disorder experience comorbid insomnia symptoms, while individuals with insomnia have more than double the risk of developing depression (Franzen & Buysse, 2008; Li, Wu, et al., 2016). Despite its high comorbidity, relatively little research has examined emotional memory processing in individuals with insomnia disorder. On the clinical side, most studies have focused on affective symptoms or comorbid emotional disorders (Baglioni, Spiegelhalder, Lombardo, & Riemann, 2010; Bottary & Denis, 2020; Hertenstein et al., 2023), overlooking emotional memory dysregulation as a distinct and potentially transdiagnostic symptom that may cut across multiple psychiatric conditions. Understanding how chronic sleep disruption, such as that observed in insomnia, impacts emotional memory development over time remains an important and underexplored area of inquiry.

It is possible that the relationship between insomnia symptoms and abnormal emotional memory processing is complex and dynamic, involving potential bidirectional influences. From a clinical perspective, abnormal emotional processing – such as maladaptive emotion regulation and negative attentional bias, commonly observed in mood-related disorders – has been shown to disrupt sleep and contribute to the development of insomnia symptoms (Vandekerckhove & Wang, 2017; Zheng et al., 2019). Conversely, a growing body of evidence suggests that chronic sleep disruption and poor sleep quality, such as that seen in insomnia, could causally impact the onset, maintenance, and worsening symptoms of psychiatric disorders characterized by dysregulated emotional memory processing, including posttraumatic stress disorder (PTSD) and depression (Gehrman et al., 2013; Koffel, Polusny, Arbisi, & Erbes, 2013; Porcheret et al., 2020; Short, Boffa, Wissemann, & Schmidt, 2020). From a mechanistic standpoint, maladaptive negative memory processing may manifest through increased pre-sleep rumination and arousal, which further increase sleep difficulty and contributing to insomnia (Lemyre, Belzile, Landry, Bastien, & Beaudoin, 2020; Tang, Saconi, Jansson-Fröjmark, Ong, & Carney, 2023). Meanwhile, individuals with insomnia are more likely to engage in increased ruminations that may reinforce negative memory biases/processing (Lewis, Taubitz, Duke, Steuer, & Larson, 2015), further exacerbating sleep problems and amplifying mood symptoms. This interplay highlights a potential self-reinforcing cycle in which poor sleep and maladaptive emotional memory processing mutually sustain and exacerbate one another. It also underscores the need to begin disentangling this complex relationship by characterizing how emotional memory processing differs between individuals with chronic sleep disruption – such as those with insomnia disorder – and healthy sleepers, as undertaken in this study.

Few studies to date have investigated how chronic sleep disruption affects emotional memory development in controlled laboratory conditions. Preliminary evidence suggested that individuals with insomnia tend to preserve negative emotional memories, as indicated by both memory performance and heightened emotional reactivity (Chunhua, Jiacui, Xue, & Kai, 2019; Wassing, Benjamins, et al., 2019). Neuroimaging findings further indicate that individuals with insomnia show sustained engagement of emotion related brain regions – particularly the dorsal anterior cingulate cortex (dACC) – when recalling past self-conscious emotional experiences, suggesting a difficulty in downregulating the emotional salience of previously encoded memories (Wassing, Schalkwijk, et al., 2019). Critically, this maladaptive emotion dissipation has been linked to fragmented rapid eye movement (REM) sleep, which may impede the amygdala’s ability to adapt to shameful memories during nocturnal sleep (Wassing, Lakbila-Kamal, et al., 2019). Such restless REM sleep may impair overnight distress resolution, leading to chronic hyperarousal in individuals with insomnia (Pesonen et al., 2024; Wassing et al., 2016).

Building on this evidence, Van Someren (2021) proposed a neurobiological model suggesting that insomnia may stem from a failure in the overnight adaptation of emotional memory. In healthy individuals, emotional charges of memories are typically attenuated during REM sleep via the silencing locus coeruleus (LC) – a key neural region involved in noradrenaline release (Cabrera et al., 2024). A silenced LC creates a unique neuromodulatory context for the limbic system, including the amygdala, to reprocess emotional experiences and attenuate their emotional intensity. However, among individuals with insomnia, the otherwise silent LC remains active, therefore impeding the fading of emotional tones of daytime experiences. This may consequently render insomnia disorder more susceptible to hyperarousal or emotional turbulence, thereby increasing the risk for developing anxiety, depression, or PTSD symptoms. Notably, daytime dysfunctions would in turn exacerbate insomnia symptoms at night, forming a vicious cycle (Nutt, Wilson, & Paterson, 2008; Oh, Kim, Na, Cho, & Chu, 2019). The hyperarousal model of insomnia provides insights into the comorbidity of insomnia with other psychiatric disorders, and opens up new avenues for understanding how sleep processes emotional memory (van der Heijden, van den Heuvel, van der Werf, Talamini, & van Marle, 2022; Vanek et al., 2020).

Despite these insights, how insomnia contributes to maladaptive emotional memory processing over extended periods remains poorly understood. Examining this question is crucial given the chronic nature of insomnia disorder, characterized by sleep disruption occurring at least three nights a week for a period of 3 months or longer, in contrast to an acute night of poor sleep or sleep deprivation in healthy sleepers (American Psychiatric Association, 2013). To bridge this gap, we examined the impact of sleep on the temporal development of emotional memory and its associated emotional tones in healthy participants and patients with insomnia disorder. Using high-density electroencephalography (EEG) across multiple sessions spanning 1 week, our study aimed to capture both the subjective experience and neural representation of emotional memory – providing a more comprehensive view of how emotional memories are processed and maintained across post-encoding (or pre-sleep), overnight sleep, post-sleep, and 7-day delayed sessions. The unparalleled temporal resolution of EEG enabled us to assess the neurodynamics of emotional memory processing along millisecond scales, while multivariate pattern analysis (MVPA) of EEG signals allow us to decode emotion specific neural representations (Bae & Luck, 2018; Zeng, Lin, Wang, & Hu, 2021). Together, these methodological advances would offer novel insight into how chronic sleep disruption affects emotional memory processing in insomnia and start to disentangle emotional memory dysregulation as a potential core and transdiagnostic feature underlying both insomnia and its psychiatric comorbidities.

## Methods

### Participants

We recruited 34 patients with insomnia disorder (ID) and 35 healthy controls (HC) (in total 46 female, mean age = 24.42, SD = 4.49). Sample size was determined based on similar studies examining emotional or fear memory among ID and HC (e.g. Bottary et al., 2020; Wassing, Schalkwijk, et al., 2019). Inclusion criteria required participants to be 18 to 35 years old, maintain a regular sleep schedule with bedtime before 2 am and sleep duration >6 hours per night. Exclusion criteria included: (1) any current or past neurological/psychiatric disorder; (2) any current sleep disorder (other than insomnia disorder); (3) chronic use of medication; (4) use of sleep medication during the prior 2 months; (5) shiftwork/intercontinental travels within 3 weeks including the experimental week (for details, see Supplementary Materials). A total of 932 potential participants completed online sign-up questionnaires assessing sleep quality, insomnia severity, depression, and anxiety. Those who passed initial prescreening via telephone were further invited to complete clinical interviews. Trained interviewers administered the Diagnostic Interview for Sleep Patterns and Disorders (DISP; Merikangas et al., 2014) and Mini-International Neuropsychiatric Interview (MINI; Sheehan et al., 1998) to confirm insomnia diagnosis and to screen for current psychiatric disorders. The insomnia module in DISP was adapted to align with DSM-5 criteria for insomnia disorder (American Psychiatric Association, 2013). While the MINI does not assess psychiatric history within a defined recent time frame, we additionally collected self-reported information regarding any clinically diagnosed psychiatric disorders within the past 5 years. This approach ensured that participants with current or recent (<5 years) psychiatric conditions were excluded from the insomnia group. The study was approved by the Human Research Ethics Committee of the University of Hong Kong (EA1808012).

### Procedure

Materials involved 48 Chinese pseudowords and 48 affective images (24 negative, 24 neutral, details see Supplementary Materials). Images are selected from the International Affective Picture System (IAPS; Lang, Bradley, & Cuthbert, 2008) and the Nencki Affective Picture System (NAPS; Marchewka, Żurawski, Jednoróg, & Grabowska, 2014).

**Figure 1.**
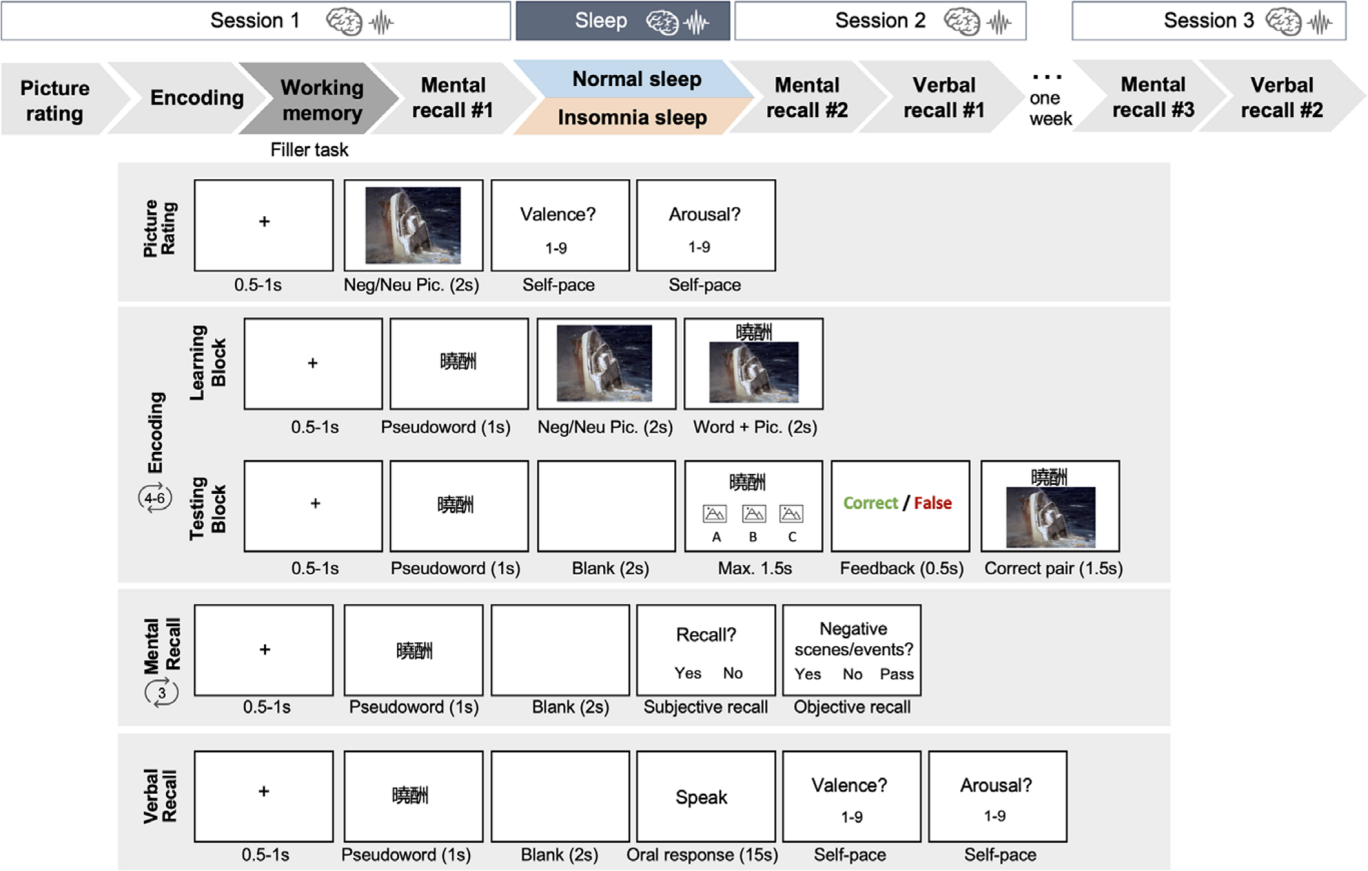
Experimental design and task details. After the adaptation night, participants came back for the experimental sessions. During all the laboratory sessions, EEG brainwaves were recorded. Encoding task was composed of learning blocks and testing blocks. One learning block was followed by one testing block (i.e. one run of study). To make sure participants fully encoded the word – picture pairs, they needed to complete at least four runs of study and to reach 90% accuracy within these four runs. For those who did not reach this 90% criterion, they would need to complete two more runs to reach an accuracy rate of at least 80%. In mental recall tasks, participants needed to mentally retrieve the affective pictures given the pseudo-Chinese words and answered two recall questions. To reach higher signal-to-noise ratios for EEGs, participants were required to recall 3 runs.

Participants completed three experimental sessions across 1 week, including two consecutive overnight stays (an adaptation night and an experimental night) at the sleep laboratory. Brain activity was recorded using a 64-channel EEG system (Eego mylab, ANT Neuro, the Netherlands) during wakeful tasks and two overnight sleep sessions (see Figure 1).

#### Lab session 1

In the experimental night, participants first rated the 48 negative and neutral pictures for valence and arousal on a 9-point Self-Assessment Manikin (SAM) scale (Bradley & Lang, 1994). They then encoded the 48 pseudoword + picture pairs through 4 – 6 learning and testing blocks, with the pseudowords randomly paired with the pictures for each participant (Figure 1 and Supplementary Materials).

Following encoding and a filler 2-back working memory task, participants completed the post-encoding mental recall task. The same mental recall task was completed three times: post-encoding, post-sleep, and during the 7-day delayed session (Figure 1). During this task, participants were prompted by the pseudoword memory cues to mentally retrieve the corresponding picture memories. They then reported whether they successfully recalled the picture (subjective recall: Yes/No), and whether the recalled picture contained negative contents/scenes (objective recall: Yes/No/Pass). To enhance the EEG signal-to-noise ratio, each recall task was repeated across three runs.

#### Lab session 2

After an 8-hour nocturnal sleep opportunity, participants completed the post-sleep mental recall task and a verbal recall task. During the verbal recall, participants were prompted by the pseudoword cues and verbally described the associated pictures in as much detail as possible. Verbal recall responses were recorded and scored by two independent raters for identification and gist scores. Identification assessed whether the participants correctly recalled the picture or not (i.e., 1/0). For gist, general gist templates were created for each picture (numbers of gists ranging from 2 to 4), and the percentage of gist elements mentioned in the participants’ description was calculated (see Supplementary Materials). Participants then rated the valence and arousal of their memories.

#### Lab session 3

Seven days later, participants completed the third mental recall task and the second verbal recall task with EEG recordings. They were then debriefed and compensated.

### Statistical analysis

Behavioral statistical analyses were performed using R version 4.2.2 (R Core Team, 2020). Sensitivity analyses were conducted by excluding participants whose average response values exceeded ±3 standard deviations from the grand mean. This exclusion was applied at the participant level to account for consistently extreme patterns across trials. The number of participants being excluded in each task and group is presented in Supplementary Table S4. Unless otherwise stated, the analyses with or without outliers yielded similar results. Detailed descriptions of model formulas are provided in the Supplementary Materials.

All behavioral analyses were conducted at the block or trial level using regression models appropriate to the outcome type. Specifically, linear mixed regression was used for continuous outcomes (e.g. encoding accuracy, memory performance from the mental recall task, gist score from the verbal recall task), mixed ordinal logistic regression for ordinal ratings (e.g. valence or arousal ratings) and mixed binominal logistic regression for binary outcomes (e.g. identification score from the verbal recall task).

The basic model included the fixed factors Group (HC vs. ID), Emotion (Negative vs. Neutral), Time/Block (when applicable), and their interactions. Participants and individual stimuli were used as random intercepts when appropriate. The full model extended the basic model by incorporating additional covariates including sex, age, written language (cue words were presented in either Traditional or Simplified Chinese, depending on the participants’ regional backgrounds), and questionnaire scores (see Supplementary Materials). To address potential multicollinearity, generalized variance inflation factors (GVIFs) were calculated, particularly given that several questionnaires may capture overlapping constructs that distinguish the healthy and insomnia groups. Two measures, the Insomnia Severity Index (ISI; Morin, Belleville, Bélanger, & Ivers, 2011) and the Pittsburgh Sleep Quality Index (PSQI; Buysse, Reynolds, Monk, Berman, & Kupfer, 1989) were excluded from the full model, as their GVIF-adjusted values exceeded a conservative threshold of 2. Additional details are provided in the Supplementary Materials.

Model selection between full and basic model was conducted using likelihood ratio tests. If the full model did not significantly improve model fit, the more parsimonious basic model was selected; otherwise, the full model was reported. Pairwise comparisons were conducted using the *emmeans* package in R (Lenth, 2020) to further examine significant interactions identified in the linear mixed effects models, allowing us to determine which fixed factor was driving the interaction effects.

### EEG acquisition, preprocessing, and analyses

EEG was recorded using a 64-channel cap (10 – 5 International System; Oostenveld & Praamstra, 2001), with an additional electrooculogram (EOG) channel. During overnight sleep, muscle activity was recorded with two additional electromyogram (EMG) electrodes attached to participants’ chin and one additional channel as EMG ground. EEG data were recorded at a 500-Hz sampling rate, referenced to CPz, with impedance maintained below 20 kΩ.

Wake EEGs were preprocessed using EEGLAB/ERPLAB (Delorme & Makeig, 2004; Lopez-Calderon & Luck, 2014) in MATLAB (MathWorks, Natick, MA). Data were downsampled to 250 Hz, band-passed (0.1 – 30 Hz), and notch-filtered (50 Hz). Re-referencing was applied using the common average. Event-related potentials (ERPs) during recall tasks were segmented (−500 to 3000 ms), with independent component analysis (ICA) used for artifact removal. Artifacts were rejected using 200 ms moving windows with a 100-ms step size when peak-to-peak amplitude exceeded ±100 microvolts. Baseline correction was applied (averaged amplitude of 500 ms prestimulus). Participants with >20% artifact trials were excluded (number of participants being excluded ranges from 0 to 3 per group, see Supplementary Table S7 for details).

To examine emotion specific neural representations, we conducted ERP-based multivariate pattern classification (i.e. decoding) on the emotion (i.e. negative vs. neutral) in healthy and insomnia groups with customized MATLAB scripts (Bae & Luck, 2018; Zeng et al., 2021). We applied a binary support vector machine (SVM) classifier to decode emotional neural representations (negative vs. neutral) using preprocessed ERPs from 61 electrodes. Decoding was performed for the picture rating (−200 to 2000 ms, for results see Supplementary Materials) and mental recall tasks (−200 to 3000 ms) using a fourfold cross-validation approach. Trials were randomly divided into four sets, with three used for training and the fourth for testing, iterating across all sets and repeated 50 times for robustness. To examine temporal dynamics, decoding was conducted on mental recall tasks both within and between sessions (post-encoding, post-sleep, and 7-day delayed recall), generating a 3 × 3 matrix of decoding accuracies. Higher between-session accuracy indicated greater similarity in neural representations across time points. Significant decoding clusters were identified using a cluster-based permutation approach (10,000 iterations, α = 0.05) in MATLAB’s FieldTrip toolbox (Oostenveld, Fries, Maris, & Schoffelen, 2011). Details can be found in the Supplementary Materials.

Sleep EEG analyses were conducted using EEGLAB/ERPLAB, and Danalyzer toolboxes (Denis, 2021) in MATLAB (MathWorks, Natick, MA). Overnight EEG data were preprocessed into low-density arrays for scoring, with selected electrodes re-referenced to the contralateral mastoids. EMG was recorded using bipolar electrodes, measuring electrical activity as the voltage difference between two closely placed electrodes. Band-pass filters (0.3 – 35 Hz for EEG, 10 – 100 Hz for EMG) and a 50-Hz notch filter were applied. A registered polysomnographic technologist (X.L.), blinded to group status, performed sleep scoring following American Academy of Sleep Medicine (AASM) Scoring Manual Version 2.6 (Berry et al., 2020). Arousals were identified following the AASM guidelines: “Score arousal during sleep stages N1, N2, N3, or R if there is an abrupt shift of EEG frequency including alpha, theta and/or frequencies greater than 16 Hz (but not spindles) that lasts at least 3 seconds, with at least 10 seconds of stable sleep preceding the change. Scoring of arousals during REM requires a concurrent increase in submental EMG lasting at least 1 second”. Extracted sleep metrics included time in bed (TIB), total sleep time (TST), sleep onset latency (SOL), wake after sleep onset (WASO), sleep efficiency (SE), time spent in N1/N2/N3/REM (in minutes and percentage), and the arousal index.

## Results

Demographical information from the two groups of participants is presented in Supplementary Table S2. Participants with ID had higher scores than HC in Insomnia Severity Index (ISI; Morin, Belleville, Bélanger, & Ivers, 2011), Pittsburgh Sleep Quality Index (PSQI; Buysse, Reynolds, Monk, Berman, & Kupfer, 1989), Beck Depression Inventory-II (BDI-II; Beck, Steer, & Brown, 1996; HC: 1.89 ± 2.61; ID: 13.65 ± 9.35), Beck Anxiety Inventory (BAI; Beck, Epstein, Brown, & Steer, 1988; HC: 2.17 ± 3.05; ID: 11.32 ± 7.95), Ford Insomnia Response to Stress Test (FIRST; Drake, Richardson, Roehrs, Scofield, & Roth, 2004), and Pre-Sleep Arousal Scale (PSAS; Nicassio, Mendlowitz, Fussell, & Petras, 1985; *p*s < 0.016). These variables were used as covariates in the behavioral analyses except for ISI and PSQI, which were excluded due to multicollinearity.

### Sleep characteristics

Sleep data from self-reported diaries and overnight EEG recordings are summarized in Supplementary Table S3. Based on sleep diary, while both groups had comparable TIB, ID reported lower TST and SE, along with longer SOL and greater WASO compared to HC. EEG-based sleep scoring confirmed a significantly longer objective SOL in ID compared to HC (*p* = 0.030), with no significant differences for other sleep macrostructure measures (*p*s > 0.058).

### Memory performance

Regression analyses on encoding accuracy suggested that the participants with ID initially outperformed HC during the first testing block (*t*(86.8) = 2.06, *p* = 0.042), though both groups achieved similar performance by the final block (for detailed results see Supplementary Materials). To account for the first block performance differences, additional model selection was performed incorporating encoding accuracy from the first block as a covariate.

## Participants with insomnia showed better memories at 7-day delay

**Figure 2.**
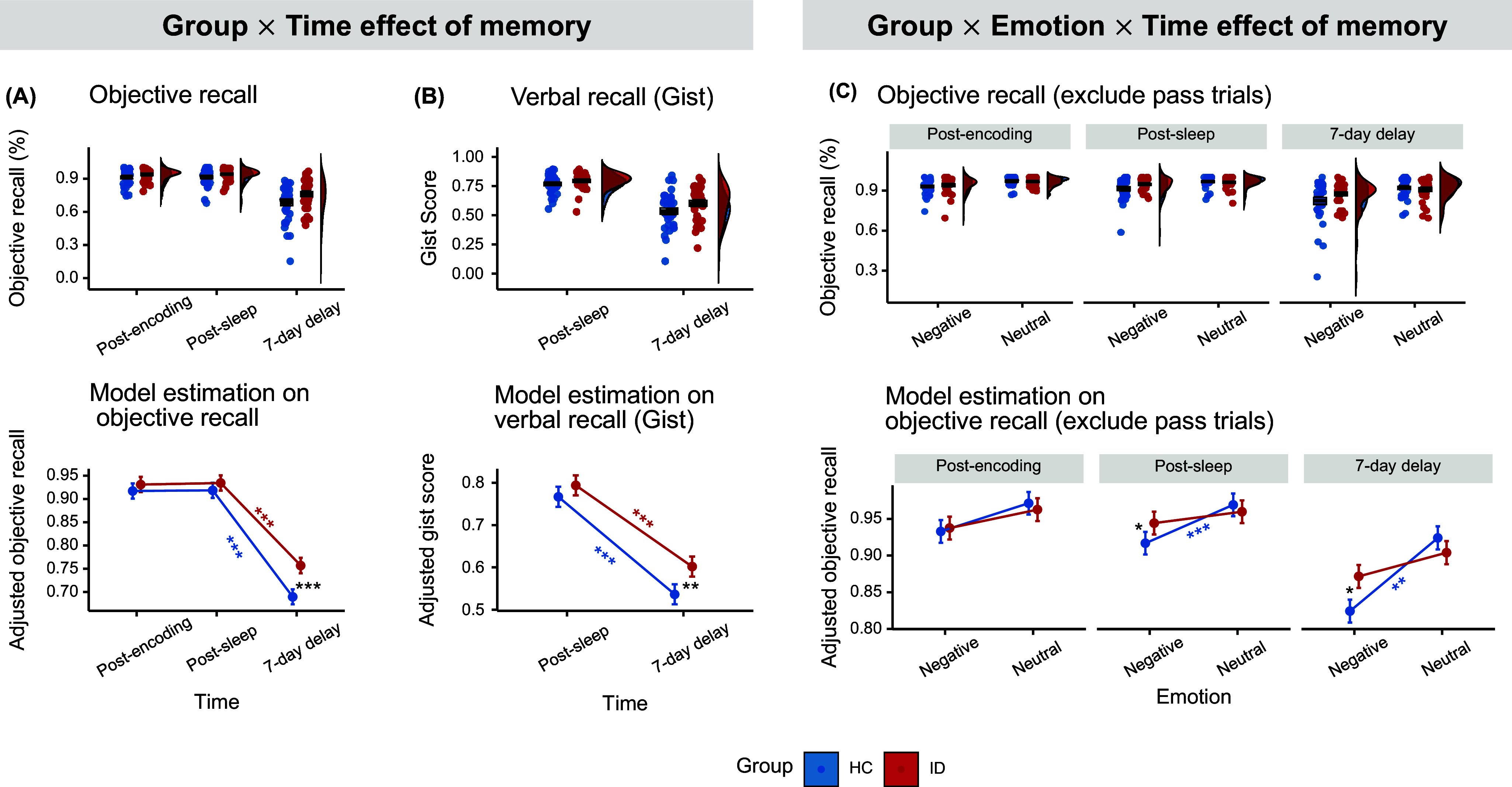
Memory results – Group 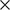 Time and Group 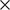 Emotion 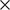 Time interaction. The Group × Time effect on objective recall from the mental recall tasks (A) and gist scores from the verbal recall tasks (B). Compared to insomnia group, healthy group showed much more memory decay from postsleep to 7-day delay, resulting in a significant group difference at 7-day delay session. (C) The Group × Emotion × Time interaction on objective recall without including ‘pass’ trials. Upper panel shows violin plot exhibiting the density of the data distribution at participant level. The box plot showed the mean (middle bold line) and 1 standard error (upper and lower boundary). Lower panel shows interaction effects extracted from the regression at trial level. *p < 0.05, **p < 0.01, ***p < 0.001.

Examining the memory performance across the two groups over post-encoding, post-sleep, and 7-day delayed sessions, we found a significant Time effect and a Group × Time interaction emerged across multiple memory indices in the mental recall tasks, including subjective recall accuracy (Time: 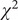 (2) = 2139.25, *p* < 0.001, 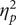 = 0.18; Interaction: 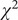 (2) = 43.12, *p* < 0.001, 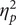 = 0.004; Supplementary Figure S2), objective recall (Time: 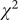 (2) = 1238.43, *p* < 0.001, 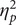 = 0.11; Interaction: 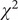 (2) = 20.77, *p* < 0.001, 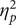 = 0.002; Figure 2a), and combined subjective and objective recall measures (Time: 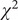 (2) = 1731.46, *p <* 0.001, 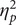 = 0.15; Interaction: 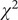 (2) = 27.14, *p <* 0.001, 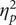 = 0.003; Supplementary Figure S2). Furthermore, significant effects of Time and Group *×* Time interactions were also observed for gist scores in verbal recall tasks (*F*(1, 6408) = 694.19, *p* < 0.001, 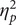 = 0.09; *F*(1, 6408) = 5.78, *p* = 0.016, 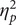 < 0.001; see Figure 2b).

Central to our research question on the temporal changes of emotional memories, while both groups had a significant memory decline from post-sleep to 7-day delayed session (see Figures 2a,b), ID showed significantly less memory decay than healthy group, resulting in a significant group d ifferences at the 7-day delayed session (*p*s < 0.009; Cohen’s *d* = 0.25 – 0.39), but not at post-encoding (*p*s > 0.137) or post-sleep (*p*s > 0.271). Sensitivity analyses largely confirmed the robustness of these results (*p*s *<* 0.008), except for the Group × Time interaction on the gist scores, which became insignificant.

## Participants with insomnia were more accurate in negative memory judgments

Furthermore, a significant Group × Emotion interaction effect was found for objective recall accuracy ( 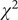 (1) = 4.61, *p* = 0.032, 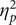 < 0.001; Supplementary Figure S3). Pairwise comparisons suggested that ID showed higher accuracy than HC for negative memory cues (*z* = 2.30, *p =* 0.022, *d* = 0.16), but not for neutral memory cues (*z* = 1.09, *p =* 0.277, *d* = 0.08). Within-group differences between negative and neutral memories were not significant in both HC (*z* = −1.46, *p =* 0.145, *d* = −0.10) and ID (*z* = −0.24, *p =* 0.808, *d* = −0.02). However, excluding outliers (±3SD) rendered the Group × Emotion effect insignificant (*p* = 0.118).

To test robustness, an additional analysis excluded ‘pass’ responses (i.e. cases where memory valence was completely forgotten). Thus, incorrect recall reflected only incorrect judgments (e.g. categorizing negative cues as associating with neutral pictures or vice versa). This analysis confirmed significant Emotion ( 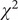 (1) = 6.14, *p* = 0.013, 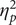 = 0.12), Time ( 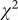 (2) = 224.70, *p* < 0.001, 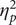 = 0.02) main effects, as well as a Group × Emotion ( 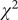 (1) = 21.76, *p* < 0.001, 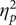 = 0.002) and a three-way Group × Emotion × Time interaction ( 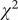 (2) = 6.81, *p* = 0.033, 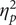 < 0.001, see Figure 2c). The Group × Emotion effect suggests that ID outperformed HC in recalling negative memories (*z* = 2.43, *p* = 0.015, *d* = 0.13), but not for neutral memories (*z* = −1.17, *p* = 0.244, *d* = −0.06).

Decomposing the three-way interaction revealed that the significant Group × Emotion effects was observed at post-sleep (*χ^2^*(1) = 9.28, *p* = 0.002, 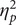 = 0.003) and delayed sessions (*χ^2^*(1) = 11.61, *p* < 0.001, 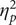 = 0.004), but not at postencoding (*χ^2^*(1) = 1.37, *p* = 0.241, 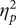 < 0.001; Figure 2c). For negative memories, a significant Group × Time interaction ( 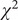 (2) = 7.48, *p* = 0.024, 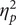 = 0.002) emerged: ID showed less memory decay than HC from post-sleep to delayed sessions, and they outperformed HC at the delayed session (*z* = 2.46, *p* = 0.014, *d =* 0.21).

Excluding outliers (±3 SD) retained a significant Group × Emotion effect ( 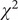 (1) = 18.78, *p* < 0.001, 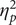 = 0.002), but rendered the Group × Emotion × Time effect nonsignificant ( 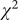 (2) = 2.61, *p* = 0.271, 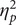 < 0.001), suggesting that while group differences in emotion-related recall were robust, their interaction with time may be influenced by outliers.

Overall, compared to HC, ID group exhibited higher accuracy in judging negative memory cues.

## The product of SWS and REM predicted negative memory decay in healthy sleepers

The significant Group × Time interactions on memory accuracy revealed differing trajectories of memory decay between ID and HC. To explore how post-encoding sleep predicts overtime memory decay (post-sleep minus 7-day delayed scores), we conducted participant-level analyses. Memory decay was calculated for negative and neutral cues on subjective, objective, and combined recall responses from the mental recall tasks, as well as gist scores from verbal recall tasks, wherein significant Group × Time effects were observed. Sleep metrics included conventional macrostructures (e.g. TST, SE, WASO, sleep stage proportions, arousal index) and a composite SWS × REM index to reflect their combined involvement (Mednick, Nakayama, & Stickgold, 2003; Stickgold, Whidbee, Schirmer, Patel, & Hobson, 2000). Spearman’s correlation analyses were conducted, with FDR correction for multiple comparisons (*n* = 9 per behavioral outcome and group; Benjamini & Hochberg, 1995).

In HC, N2 proportion and SWS × REM exhibited a nonsignificant trend predicting negative memory decay, as measured by objective recall (excluding pass trials; *p*s_corrected_ = 0.054 – 0.072; see Supplementary Figure S4). These correlations became significant (*r*_s_ = −0.45 to 0.47, *p*s_corrected_ = 0.045) after excluding ±3 SD outliers (*n* = 3 and 2). Specifically, shorter N2 sleep and greater SWS × REM were associated with greater decay for negative memories over 7 days. This relationship was not observed in ID (*r*_s_ = −0.11 to 0.01, *p*s_corrected_ = 0.759 – 0.977).

Fisher’s *Z* tests confirmed that the correlation coefficients between SWS × REM and negative memory decay significantly differed between groups, both with (*z* = 1.97, *p* = 0.049, Cohen’s *q* = 0.50) and without outliers (*z* = 2.33, *p* = 0.020, Cohen’s *q* = 0.60). However, no significant between-group differences were found for N2 proportion (*p* > 0.088). Robust regression analyses controlling for individual and joint contributions of SWS and REM confirmed that SWS × REM was the strongest predictor of negative memory decay ( 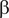 = 0.001, *p* = 0.035), suggesting in HC, higher SWS × REM was uniquely associated with greater negative memory decay over a week, while this pattern was absent among participants with ID.

## Affective ratings during verbal recall tasks

### Participants with insomnia exhibited impaired overtime emotion dissipation

**Figure 3.**
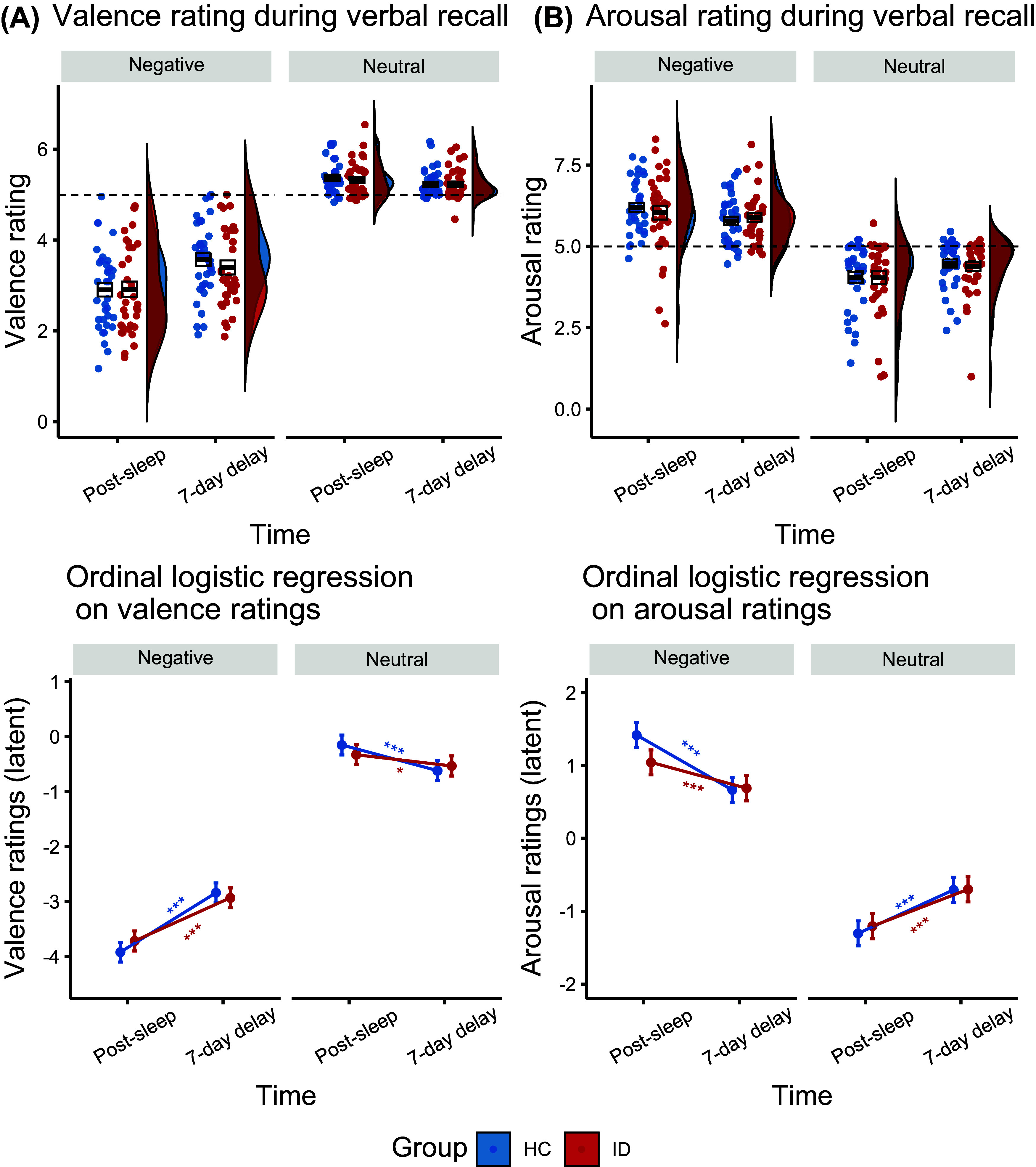
Emotional ratings from verbal recall tasks. (A) Valence and (B) arousal rating at verbal recall across groups and time. Upper panel shows violin plot showed the density of the data distribution at participant level. The box plot showed the mean (middle bold line) and 1 standard error (upper and lower boundary). Lower panel shows interaction effects extracted from the ordinal logistic regression at trial level. *p < 0.05, **p < 0.01, ***p < 0.001.

In addition to memory performance, we were also interested in the subjective emotional response associated with the memory. After controlling for baseline affective ratings (baseline results see Supplementary Materials), we observed a significant three-way Group × Time × Emotion interaction for both valence ( 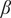 = −0.07, 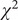 (1) = 7.67, *p* = 0.006) and arousal ( 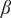 = 0.06, 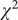 (1) = 6.85, *p* = 0.009; see Figure 3). Further analyses revealed a significant Group × Time for negative (valence: 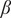 = −0.08, 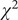 (1) = 5.86, *p* = 0.016; arousal: 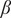 = 0.10, 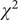 (1) = 9.59, *p* = 0.002), but not for neutral memories (*p*s > 0.083). While both groups exhibited significant affect attenuation for negative memory over time (i.e. less negative and less arousing), the effect was significantly stronger in HC (valence: *z* = 13.22, *p* < 0.001, *d* = 0.51; arousal: *z* = −8.41, *p* < 0.001, *d* = −0.33) compared to ID (valence: *z* = 9.99, *p* < 0.001, *d* = 0.38; arousal: *z* = −4.07, *p* < 0.001, *d* = −0.16). No group differences were found at either the post-sleep or the 7-day delayed session (*p*s > 0.294). These results indicated that while HC exhibited greater emotion dissipation over time, this process was impaired in ID, as exhibited by a significantly smaller reduction.

## REM proportion predicted arousal reduction for negative memory in healthy group

Based on the behavioral results, we calculated overtime emotional changes (i.e. 7-day delayed minus post-sleep valence/arousal ratings) for negative and neutral cue words and conducted Spearman’s correlation with post-encoding sleep metrics with FDR corrections.

In HC, greater overtime arousal reduction was significantly correlated with higher REM proportion (*r*_s_ *=* −0.45, *p*_corrected_ = 0.027), lower N2 proportion (*r*_s_ *=* 0.44, *p*_corrected_ = 0.027), and larger SWS × REM (*r*_s_ *= −*0.45, *p*_corrected_ = 0.027; see Supplementary Figure S5). Yet these patterns were absent in ID (*r*_s_ *=* −0.25 to 0.26, *p*s_corrected_ = 0.495). Removing ±3 SD outliers did not affect the significance (*p*s_corrected_ = 0.048). However, Fisher’s *Z* tests did not reveal significant between-group differences in these correlation coefficients (*p*s > 0.23, Cohen’s *q* = −0.31 to 0.245). Further robust regression analyses controlling for individual and joint contributions of SWS and REM revealed that only higher REM proportion predicted greater overtime arousal reduction among HC ( 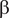 = −0.03, *p* = 0.041), highlighting REM sleep’s critical role in emotional dissipation.

## Tracking neural representation of emotional memory over time

Overall, the emotion related ERP (see Supplementary Figures S10 and S11), for example, P300 and LPP, revealed limited between-group differences. We next conducted MVPA using whole-brain EEG to examine the neurodynamics of emotional memory processing within and across sessions. Specifically, we conducted multivariate time-resolved EEG decoding analyses across the three mental recall tasks to assess whether scalp EEG activity patterns could differentiate between negative and neutral memories, and to track how emotion related neural representations evolved over time – namely, following encoding, after sleep, and at a 7-day delayed session – in ID and HC groups. Given the robustness of the results across measures, we reported correct objective recall trials (i.e. trials where participants made correct negative/neutral judgment based on the cue words; see Supplementary Figure S12 for combined subjective and objective recall results).

**Figure 4.**
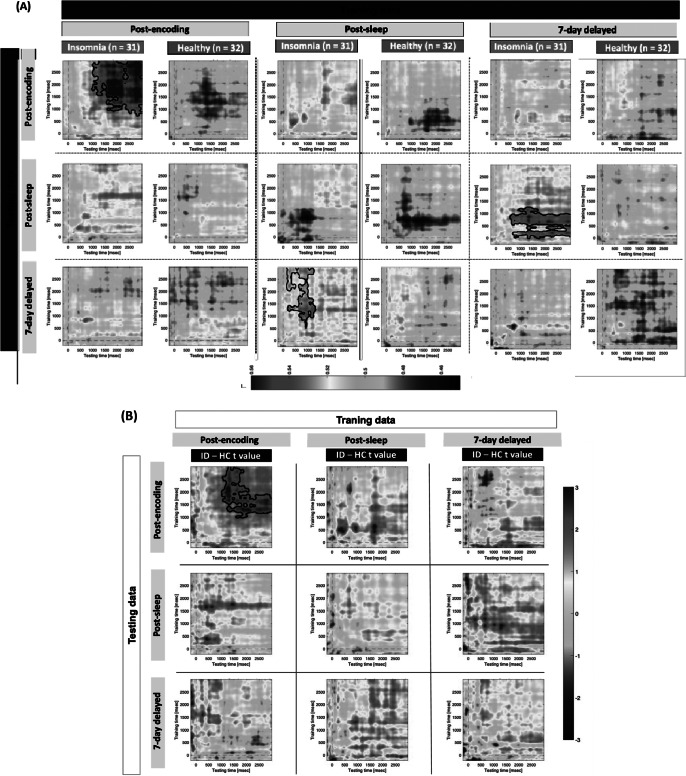
Multivariate pattern analysis for mental recall tasks based on correct objective recall trials. We conducted ERP-based multivariate pattern analysis (MVPA) to determine whether whole-brain EEG activity could distinguish between emotional valence (negative vs. neutral) during mental recall tasks, and how these neural patterns evolved across sessions in participants with insomnia disorder (ID) and healthy controls (HC). (A) Time – time decoding matrices for each group across sessions. Each matrix depicts decoding accuracy across all pairwise time points – training time (*y*-axis) and testing time (*x*-axis) – within or between sessions. A decoding value above the chance level (0.5) indicates that the EEG signal contains information that discriminates negative from neutral memory cues. Significant clusters (black contours) mark time regions where decoding accuracy was significantly greater than chance (*p* < 0.05, two-tailed, cluster-corrected). Within-session decoding (diagonal panels) reflects emotion specific representations during the same session; between-session decoding (off-diagonal panels) assesses whether emotional representations are preserved across sessions. (B) Group-level differences in decoding accuracy (ID minus HC) for each time – time matrix, corrected via nonparametric permutation testing. Significant clusters (outlined in black) reflect time points where decoding accuracy differed reliably between groups (*p* < 0.05, two-tailed). The use of both axes to represent time allows for examination of temporal generalization – that is whether EEG patterns trained at a specific time point can generalize across other time points, within or across sessions. HC = healthy controls; ID = insomnia disorder; ERP = event-related potential.

Within-session decoding suggested that only ID showed significant above-chance decoding accuracy during post-encoding recall (740 – 3000 ms, corrected *p* = 0.027, 10,000 permutations; Figure 4a). Moreover, ID showed significantly higher decoding accuracies than HC (1 cluster, corrected *p* = 0.046, see Figure 4b).

Between-session decoding examined whether neural representations at a specific session can be generalized to other sessions. Again, only ID showed above-chance decoding clusters: classifiers trained on post-sleep recall at around 560 – 3000 ms successfully decoded neural representations from 7-day delayed recall at around 140 – 1300 ms (corrected *p* = 0.045, see Figure 4a), and vice versa (classifiers trained on delayed recall from 20 to 1280 ms decoded post-sleep recall from 560 to 3000 ms, corrected *p* = 0.028). However, between-group comparisons showed no significant differences.

Collectively, multivariate decoding results suggested that during mental recall, participants with ID exhibited robust emotion-related neural representation during the post-encoding session. In contrast, HC did not show such patterns at any time point. Furthermore, among the ID group, emotional memory representations observed during the post-sleep session closely resembled those at the 7-day delayed session, as indicated by significant cross-session decoding clusters. These results suggested that ID retained a stable neural representation of emotion over time, a pattern not observed in HC.

## Discussion

We examined how chronic sleep disruption impacts the temporal development of emotional memory by comparing individuals with insomnia disorder to healthy sleepers across 1 week of behavioral and EEG-based assessments. Results suggested that individuals with insomnia exhibit impaired dissipation of emotional memory over time, both subjectively and neurally, in contrast to healthy sleepers who showed adaptive emotional memory fading. Notably, in healthy sleepers, the product of SWS and REM sleep predicted greater decay of negative memory, with REM sleep predicting greater arousal reduction for negative memory over the week. In sharp contrast, these relationships were absent in participants with insomnia, suggesting that abnormal SWS and REM sleep may contribute to the maladaptive reprocessing of emotional memories over time.

Participants with insomnia demonstrated better memory performance than healthy sleepers at the 7-day delayed recall across multiple measures. This retention superiority was primarily driven by a greater retention of negative memory in the insomnia group. These findings align with a previous study showing that individuals with insomnia had impaired recognition of positive and neutral images, but enhanced retaining of negative image recognition – indicating a selective retention of negative emotional memories despite broader recognition deficits (Chunhua, Jiacui, Xue, & Kai, 2019). Similar patterns have been observed in studies of sleep-deprived individuals and those with poor sleep quality, who also show preferential retention of negative content over time (Cellini, Mercurio, & Sarlo, 2019; Sterpenich et al., 2007; Zeng et al., 2021). Together, these converging results suggest that both acute and chronic sleep disturbance would selectively preserve negative memory.

In contrast, healthy individuals exhibited a significant negative memory decay over time (Zeng et al., 2021). In particular, longer post-encoding SWS and REM sleep (as measured by the product of SWS and REM proportions) predicted greater forgetting of negative memories over 1 week, a relationship absents in individuals with insomnia. These findings extend prior actigraphy-based work linking higher sleep efficiency to increased forgetting of negative stimuli, providing fine-grained EEG evidence for the synergistic role of SWS and REM sleep (Cellini et al., 2019). Building upon the sequential hypothesis of sleep-dependent memory consolidation (Ambrosini & Giuditta, 2001; Ambrosini, Sadile, Gironi Carnevale, Mattiaccio, & Giuditta, 1988), SWS and REM sleep play complementary roles in processing newly acquired information (Cairney, Durrant, Power, & Lewis, 2015; Hu et al., 2015; Stickgold et al., 2000). Complementing the synaptic homeostasis hypothesis that posits that REM sleep would facilitate forgetting via pruning and renormalizing synaptic activity, our findings suggest that SWS may also play an essential role in adaptive dissipation of aversive memories by setting the stage for subsequent REM-mediated processing. Supporting this idea, an fMRI study found that longer SWS duration was linked with reduced hippocampal activity during the recollection of negative memories. The subsequent REM sleep was linked to increased functional connectivity between the hippocampus and frontal regions during the same recollection task (Cairney et al., 2015). These findings collectively point to a sequential and complementary role of SWS and REM sleep in facilitating the attenuation of negative memories in healthy sleepers.

When recalling negative pictures, healthy sleepers showed a marked reduction in both the perceived negativity and arousal of their memories over a week, along with an absence of between-session neural similarities, that is adaptive emotional dissipation. In contrast, individuals with insomnia exhibited a significantly smaller trend in overtime affect reduction and a sustained emotion-specific neural representation, indicating impaired emotion dissipation. These findings align well with the ‘Sleep to Forget’ hypothesis and with previous research, where adequate sleep attenuates affective responses to negative stimuli over time (Bolinger et al., 2019; Cunningham et al., 2014; Goldstein & Walker, 2014; Werner, Schabus, Blechert, & Wilhelm, 2021; Zeng et al., 2021). Conversely, participants with insomnia were unable to resolve past emotions, exhibiting an impaired ‘Sleep to Forget’ effect (Van Someren, 2021). Indeed, when individuals with insomnia recalled remote shameful experience, they engaged emotion specific neural circuits (e.g. dorsal ACC) highly overlapping with the circuits engaged when experiencing new shameful experiences. In contrast, when healthy participants recalled remote emotional events, they did not show limbic region responses, indicating successful emotion dissipation (Wassing, Schalkwijk, et al., 2019). These findings collectively suggested that while healthy sleep promotes overtime emotion dissipation, disrupted sleep in insomnia may lead to impaired dissipation and sustained neural representations of emotion.

Notably, among healthy individuals, higher REM proportion during the post-encoding sleep was correlated with greater overtime arousal reduction. However, this pattern was again, absent among individuals with insomnia, though the group differences did not reach a statistical significance. Our results are consistent with a recent study suggesting that increased REM duration was associated with stronger overnight reduction of amygdala activation toward shameful experiences (Wassing, Lakbila-Kamal, et al., 2019). Furthermore, disrupted REM sleep, as commonly seen in insomnia, would abolish this beneficial effect on emotion adaptation (Feige et al., 2008; Wassing, Lakbila-Kamal, et al., 2019). Interestingly, we did not observe heightened levels of sleep fragmentation or arousal in the insomnia group (Supplementary Table S3). While further research is needed to explore REM interruptions in individuals with chronic sleep disturbances, these findings underscore the crucial role of consolidated REM sleep in emotional adaptation.

Limitations and future directions shall be discussed. First, the relationships between SWS × REM and REM proportion with memory/emotion outcomes were based on correlations. Future research would benefit from establishing the causal role of SWS/REM sleep and memory reactivation in adaptive forgetting of negative memories (Hu, Cheng, Chiu, & Paller, 2020; Hutchison, Pezzoli, Tsimpanouli, Abdullah, & Lewis, 2021). Second, our results were based on the recall tasks, which could be different from the recognition paradigms that involve repeated exposure to emotional stimuli. It is worth emphasizing that variations in task designs (e.g. recall vs. recognition) may influence outcomes and can be related to replication challenges in emotional memory research (Cunningham, Stickgold, & Kensinger, 2022). Third, this study was conducted among young adults with primary insomnia disorder and without any current or recent psychiatric disorders. Thus, the conclusions may not be generalizable to individuals with comorbid insomnia and psychiatric disorders or those in different age groups. Future research should further examine how comorbidities and age differences may affect sleep in processing emotional memory.

To conclude, our results reveal distinct temporal trajectory of emotional memory development in individuals with insomnia disorder compared to healthy sleepers. Behavioral and neural measures collectively suggest that individuals with insomnia disorder exhibited impaired emotional memory dissipation over time. Among healthy sleepers, both SWS and REM sleep contributed to the adaptive emotional memory dissipation. By using insomnia as a real-world model of chronic sleep disruption, our study delineates how emotional memory unfolds and persists over time. The longitudinal, laboratory-controlled tracking of emotional memory dynamics over a full week represents a methodological advance in understanding the cumulative impact of sleep on emotional memory. These results bear important translational implications – not only for the prevention and treatment of insomnia itself, but also for a broader range of psychiatric conditions in which insomnia symptoms are common. For instance, disrupted sleep may hinder patients with PTSD from adaptive processing of traumatic experiences. Our results highlight the potential for sleep-targeted interventions to facilitate emotional memory resolution, offering a promising path for improving emotion regulation and mental health across clinical populations.

## Supporting information

Zeng et al. supplementary material Zeng et al. supplementary material

## Data Availability

The data and software/scripts used in this study are available on reasonable request from the corresponding author.

## Supplementary material

The supplementary material for this article can be found at http://doi.org/10.1017/S0033291725101566.
