# Supplementary material for: Impaired emotional memory dissipation in insomnia disorder
Source: Psychol Med. 2025 Sep 1;55:e260. doi: 10.1017/S0033291725101566 (PMC13040600; doi:10.1017/S0033291725101566)
Supplement: Zeng et al. supplementary material [file S0033291725101566sup001.docx]

**Supplementary Materials**

**Table of Contents**

[Screening process 2](#_Toc203489890)

[Material 3](#_Toc203489891)

[Pseudo words as memory cues 3](#_Toc203489892)

[Affective picture stimuli 3](#_Toc203489893)

[Measurement 4](#_Toc203489894)

[Encoding task 4](#_Toc203489895)

[Mental recall tasks 4](#_Toc203489896)

[Verbal recall tasks 5](#_Toc203489897)

[Behavioral analysis 5](#_Toc203489898)

[Memory 6](#_Toc203489899)

[Emotion 7](#_Toc203489900)

[EEG multivariate analysis 8](#_Toc203489901)

[Results 9](#_Toc203489902)

[Demographic descriptives 9](#_Toc203489903)

[Sleep characteristics 10](#_Toc203489904)

[Sensitivity Analyses: Participant exclusion based on ±3SD criteria 10](#_Toc203489905)

[Model Generalized Variance Inflation Factors (GVIFs) 11](#_Toc203489906)

[Encoding accuracy 12](#_Toc203489907)

[Memory performance 14](#_Toc203489908)

[Correlation analyses on sleep and memory decay 17](#_Toc203489909)

[Regression analyses on sleep and memory decay 17](#_Toc203489910)

[Baseline valence/arousal ratings from the picture rating task 17](#_Toc203489911)

[Correlation analyses on sleep and emotion dissipation 20](#_Toc203489912)

[Affective ratings accounting for memory scores during post-sleep and 7-days delayed verbal recall tasks 21](#_Toc203489913)

[ERP results 26](#_Toc203489914)

[ERP-based decoding results for recall tasks based on correct subjective and objective recall trials 31](#_Toc203489915)

[References 33](#_Toc203489916)

**Screening process**

Potential participants signed up through online questionnaires (n = 932) and completed the Insomnia Severity Index (ISI; Morin, Belleville, Bélanger, & Ivers, 2011) Pittsburgh Sleep Quality Index (PSQI; Buysse, Reynolds, Monk, Berman, & Kupfer, 1989) Beck Depression Inventory-II (BDI-II; Beck, Steer, & Brown, 1996) and Beck Anxiety Inventory (BAI; Brown & Epstein, 1988) They then underwent pre-screening telephone calls during which insomnia criteria from the Diagnostic and Statistical Manual of Mental Disorders, Fifth Edition (DSM-5; American Psychiatric Association, 2013) were briefly checked.

Pre-screened participants were then included in online clinical interviews where DISP and MINI were conducted (Merikangas et al., 2014; Sheehan et al., 1998). The insomnia module in DISP was modified and adopted to ascertain insomnia disorder according to DSM-5 criteria. The DISP was used to rule out any sleep disorders expect for insomnia, based on The International Classification of Sleep Disorders - 2nd edition (ICSD-II; American Academy of Sleep Medicine, 2005) criteria, including delayed sleep phase, restless leg syndrome, obstructive sleep apnea, hypersomnia etc. The MINI was used to rule out any current or recent (<5 years) major psychiatric disorders, e.g., major depressive disorder, anxiety disorder, post-traumatic stress disorder, psychotic disorders etc.

A total of 83 participants were deemed eligible and invited to participate in the experiments. However, 10 participants dropped out for various reasons, including COVID infection, unwillingness to undergo EEG recording, and inability to abstain from caffeine during the study period. One participant was excluded due to disrupted EEG data collection, and another withdrew after failing to meet the 90% accuracy threshold during the encoding task. Additionally, two participants were found ineligible during the study (e.g., inability to read Chinese, history of a depressive episode within the past 2–3 years). The final sample comprised 69 participants (ID = 34, HC = 35).

**Material**

### Pseudo words as memory cues

70 Chinese pseudowords were created. Twenty-three participants from an independent group rated their valence, arousal and familiarity (ratings from 1 to 9, with 1 as negative/calm/least familiar, 5 as neutral, and 9 as positive/excited/most familiar, respectively). 48 of the pseudo words were selected according to 1) averaged valence and arousal ratings are lower than 6 and higher than 4 (i.e., ensuring emotional neutrality); 2) averaged familiarity is lower than 5 (i.e., ensuring unfamiliarity, see Supplementary Table 1 for statistics).

### Affective picture stimuli

96 affective pictures (48 negative, 48 neutral; normative ratings see Supplementary Table 1) from International Affective Picture System (IAPS)(Lang, Bradley, & Cuthbert, 2008) and Nencki Affective Picture System (NAPS)(Marchewka, Żurawski, Jednoróg, & Grabowska, 2014) were selected. Forty-eight of them (24 negative, 24 neutral) were used as encoding materials and were randomly paired with the 48 pseudo words. The rest of the pictures (24 negative, 24 neutral) were used as stimuli for the new picture viewing task.

**Supplementary Table 1 Normative ratings for pseudo words and picture stimuli (mean ± SD)**

| **Pseudo words** | | | | | |
| --- | --- | --- | --- | --- | --- |
| Valence | | Arousal | | Familiarity | |
| 5.09 ± 0.42 | | 4.67 ± 0.32 | | 3.27 ± 0.50 | |
| **Picture stimuli** | | | | | |
|  | Encoding set | | New picture set | | Test |
| **Valence** |  | |  | |  |
| Negative | 2.51 ± 0.55 | | 2.61 ± 0.65 | | *t*(44.65) = -0.58, *p* = 0.56 |
| Neutral | 5.34 ± 0.31 | | 5.36 ± 0.33 | | *t*(45.79) = -0.23, *p* = 0.82 |
| **Arousal** |  | |  | |  |
| Negative | 6.15 ± 0.59 | | 6.05 ± 0.84 | | *t*(41.21) = 0.48, *p* = 0.64 |
| Neutral | 3.89 ± 0.62 | | 3.89 ± 0.84 | | *t*(42.48) = -0.03, *p* = 0.98 |

*Notes.* The normative ratings for pseudo words are from 23 additional participants, while the normative ratings of pictures were from the International Affective Picture System and Nencki Affective Picture System.(Lang et al., 2008; Marchewka et al., 2014)

**Measurement**

Eligible participants completed questionnaires including the Reduced Morningness–Eveningness Questionnaire (r-MEQ) (Adan & Almirall, 1991), Ford Insomnia Response to Stress Test (FIRST) (Gelaye et al., 2016), Pre-Sleep Arousal Scale (PSAS) (Nicassio, Mendlowitz, Fussell, & Petras, 1985), Insomnia Severity Index (ISI) (Morin et al., 2011), Pittsburgh Sleep Quality Index (PSQI) (Buysse et al., 1989), Beck Depression Inventory-II (BDI-II) (Beck et al., 1996) and Beck Anxiety Inventory (BAI) (Brown & Epstein, 1988). Participants maintained paper-based sleep diaries to log daily sleep-wake patterns and activities, starting 7 days prior to the adaptation night and continuing through the final lab session. They were instructed to avoid alcohol, caffeine, and naps for at least 24 hours before any lab sessions.

### Encoding task

Following the picture rating task, participants viewed word-picture pairings, in which 48 pseudo Chinese words were randomly paired with the 48 picture stimuli. The encoding task consisted of learning blocks, each followed by testing blocks. In the learning blocks, participants were instructed to pay full attention to and to memorize the 48 word-picture pairs. No responses were needed in the learning blocks. Following each learning block, participants finished the testing block, participants needed to choose the correct picture that being paired with the pseudo word from 3 options by pressing corresponding button with time pressure (1.5s). Note that all 3 options were chosen from the 48 pictures participants previously viewed, to ensure that participants could remember the correct pairings without relying on familiarity. Instant feedback and correct word-picture pair (1.5s) were given to enhance participants’ memory. Timely responses were emphasized, and participants were instructed to imagine the corresponding pictures in their mind as soon as they saw the pseudo words for timely responses. Participants were required to reach an accuracy of 90% within 4 to 6 rounds of learning and testing. Only one participant did not meet this criterion and was removed from the analysis.

### Mental recall tasks

Participants were tested for their cued recall memories for the 48 pairs three times throughout the study period (see main text Figure 1). In the cued recall tests, participants were presented with the pseudo word (1s), followed by a 2s blank screen for recalling the corresponding pictures. Then participants self-reported whether they successfully recalled the picture or not given the cue (i.e., subjective recall question: Yes/No), and whether the picture they recalled contained negative scenes or events (i.e., objective recall question: Yes/No/Pass) by pressing corresponding buttons. Participants were instructed to recall and imagine the corresponding pictures as soon as they saw the pseudo word cues. They were also explicitly instructed that for a “successful recall”, they should be able to recall the corresponding picture vividly and uniquely to distinguish it from the other pictures they learnt. Otherwise, they need to indicate “No” in the subjective recall question. If they could not recall anything at all, they were instructed to press “No” in the subjective recall question followed by “Pass” in the objective recall question. They were also reminded that in some cases, they may fail to recall the picture successfully, but they could still tell whether the picture contains negative scenes/events. In such cases, they could indicate “No” in the subjective question, but also indicate “Yes/No” instead of “Pass” in the objective question. Participants completed 3 rounds of recall (each contain the 48 pseudo words presented randomly) to increase the signal-to-noise ratio of EEG brainwaves.

### Verbal recall tasks

Participants needed to verbally recall the 48 pictures given the associated pseudo words. In each trial, participants viewed pseudo word for 1s and were given a 2s blank screen for recalling the corresponding picture. Then they verbally described the picture as detailed as possible for 15s. Their oral responses were recorded for later coding. Lastly, they needed to rate their emotions when recalling these pictures in terms of valence and arousal on two 9-point SAM scales.

**Behavioral analysis**

For all the behavioral analysis, we used linear mixed regression for continuous outcomes (e.g., encoding accuracy, subjective/objective recall performance, gist score etc.), mixed ordinal logistic regression for ordinal ratings (e.g., valence or arousal ratings etc.) and mixed binominal logistic regression for binary outcomes (e.g., identification score etc.). Basic model involves Group, Emotion, Time (if appropriate) and their interactions as fixed predictors, participants and individual pictures were used as random intercepts when appropriate. The full model was basic model with additional covariates including gender, age, written language, MEQ type, BDI-II, BAI, PSAS and FIRST. Model selection was completed through comparing the basic model and full model via likelihood ratio tests. If the full model did not yield significant improvement, the parsimonious model would be selected, otherwise, the full model would be reported. Specific model used in each measurement was reported in the result section correspondingly.

### Memory

Trial-level subjective and objective recall score were obtained from the three recall tasks, during post-encoding, post-sleep and 7-day delayed sessions, respectively. In each trial of the recall task, participants indicated 1) whether they could recall the corresponding picture vividly and uniquely (yes [1]/no [0]), the answer of which was coded as subjective recall score; 2) whether the picture they recalled contains any negative scenes/events (yes [1]/no [0]/pass [0]), with the answer coded as objective recall score. Note that the objective recall does not require participants to unambiguously identify the picture, but rather a binary valence judgment of the memory. Thus, even if they indicated “no” in the subjective recall, they could still indicate “yes” (i.e., contains negative scenes) or “no” (i.e., no negative scenes) in the objective recall.

Since each memory cue was presented once during each of the three blocks, we calculated the proportion of correct response for each memory cue across these three blocks. Linear mixed models were conducted: Subjective/Objective/Combined recall (% of correct recall across three blocks) ~ Group * Time * Emotion + (1|participant) + (1|picture).

The verbal descriptions for each picture recorded from the two verbal recall tasks during post-sleep and 7-day delayed session were transcribed and scored by independent raters in the following aspects:

(1) *Identification*. Binary measure (0/1) assessing whether the participant correctly recall the picture. The descriptions that were scored as 1 should be able to allow the raters to identify the exact picture uniquely and unambiguously.

(2) *Gist*. A series of general gist were created for each picture (ranging from 2 to 4). The percentage of gist mentioned in participant’s description was scored.

The standardized manual for scoring was adapted and developed based on (Küpper, Benoit, Dalgleish, & Anderson, 2014). Two raters rated each trial separately and the inter-rater reliabilities were high (Cohen’s kappa ranges from 0.970 – 0.978). For identification, if the two raters gave different scores, a third rater (SZ) would reconcile the inconsistency. For gist, the proportion of correctly mentioned gist for each picture was calculated and the averaged gist percentage of the two raters were used as the outcome variable.

Due to a technical error, one participant’s recording was lost and thus removed from the following analysis (final N = 63). Binary logistic regression and linear mixed regression were applied to trial-level identification and gist percentage respectively:

Identification (0/1) ~ Group * Time * Emotion + (1|participant) + (1|picture)

Gist (percentage) ~ Group * Time * Emotion + (1|participant) + (1|picture)

### Emotion

Using the *clmm* function from the “ordinal” R package,(Christensen, 2022) we applied mixed ordinal logistic regressions on the trial-level data to examine the ordinal valence/arousal ratings obtained during the picture rating task:

Valence/arousal ratings ~ Group * Emotion + (1|participant) + (1|picture).

During the verbal recall tests, participants also gave valence and arousal ratings to their recalled memories. We conducted trial-level mixed ordinal logistic regressions on the valence and arousal ratings with baseline ratings (i.e., ratings obtained during the picture rating task) as a covariate: Valence/arousal ratings ~ Group * Time * Emotion + Baseline ratings + (1|participant) + (1|picture).

Furthermore, to account for the influence of memory on affective ratings, we additionally added the identification score (0/1) as one of the fixed predictors and employed ordinal logistic regression to their corresponding valence and arousal ratings: Valence/arousal ratings ~ Group * Time * Emotion* Identification + Baseline ratings + (1|participant) + (1|picture).

One participant reported to misunderstand the concept of arousal and thus was removed from all arousal analysis.

**EEG multivariate analysis**

We used preprocessed ERPs from 61 electrodes and applied a binary support vector machine (SVM) classifier to decode between negative and neutral conditions at each time point within the -200 to 2,000 ms window for the picture rating task, and -200 to 3,000 ms for the mental recall tasks. We used a 4-fold cross-validation procedure: for each participant and each condition, 24 trials for the picture rating task and 72 trials for the mental recall task (if none were rejected) from each condition (i.e., negative and neutral) were randomly divided into four sets. Data were then averaged within each set to generate four sub-ERPs for both conditions. Three sub-ERPs were used for training, and the performance was tested with the fourth sub-ERP for every 100 ms sliding window to obtain temporal generalization. This procedure was repeated four times, with each of the four sets serving as the test data once, and the remaining three sets used for training. To ensure robust decoding, the procedure was repeated 50 times with different random assignments to generate the four averaged sub-ERPs. Decoding accuracy was calculated by comparing the true condition labels with the predicted labels, averaged over cross-validations and iterations.

To unravel the temporal dynamics of the emotional neural representation across the recall tasks at different timepoints (i.e., post-encoding, post-sleep and 7-day delayed sessions), the decoding procedure was conducted both within- and between-sessions. For between-session decoding, the training sets consisted of the ERPs from one recall task, while the testing sets consisted of ERPs from the recall task at a different timepoint. Higher accuracies in between-session decoding would indicate greater neural representation similarity of emotion between these two sessions (e.g., if session 1 neural activity contains similar representations as in session 2 neural activity, decoding between the sessions would be more accurate). For the healthy and insomnia groups separately, this analysis generated a 3*3 matrix representing the three same-session decoding results (i.e., training and testing conducted within post-encoding, post-sleep and delayed sessions) and six between-session decoding results (e.g., training on post-encoding and testing on post-sleep/delay session, training on post-sleep and testing on post-encoding/delay session, training on delay and testing on post-encoding/sleep session).

To identify significant decoding clusters (> chance level 0.5 for distinguishing negative vs. neutral), we used a cluster-based permutation approach with the ft_statfun_depsamplesT function in MATLAB's FieldTrip toolbox (Oostenveld, Fries, Maris, & Schoffelen, 2011). We conducted 10,000 iterations with a cluster alpha of 0.05 (two-sided tests) using the default maxsum method for cluster significance. For group comparisons, we utilized the ft_statfun_indepsamplesT function.

## Results

### Demographic descriptives

**Supplementary Table 2** **Demographic information for healthy and insomnia group (N = 69; mean ± SD)**

|  | Heathy  (N = 35) | Insomnia  (N = 34) | Test statistics |
| --- | --- | --- | --- |
| **Age** | 23.91 ± 4.47 | 24.94 ± 4.51 | *W* = 514, *p* = 0.333 |
| **Gender** |  |  |  |
| Male, n (%) | 12 (34.29%) | 11 (32.35%) |  |
| Female, n (%) | 23 (65.71%) | 23 (67.65%) | $\chi^{2}$(1) = 0.03, *p* = 0.865 |
| **Insomnia Subtype^a^** |  |  |  |
| Difficulty in initiating sleep (DIS) | NA | 20 (58.82%) |  |
| Difficulty in maintaining sleep (DMS) | NA | 1 (2.94%) |  |
| Early morning awakening (EMA) | NA | 4 (11.76%) |  |
| Mixed type | NA | 9 (26.48%) |  |
| **Insomnia Severity Index (ISI)** | 2.97 ± 2.47 | 17.15 ± 3.62 | *W* = 0.00, *p* < 0.001 *** |
| **Pittsburgh Sleep Quality Index (PSQI)** | 2.83 ± 1.60 | 9.06 ± 2.45 | *W* = 5.50, *p* < 0.001 *** |
| **Beck Depression Inventory-II (BDI-II)** | 1.89 ± 2.61 | 13.65 ± 9.35 | *W* = 56.00, *p* < 0.001 *** |
| **Beck Anxiety Inventory (BAI)** | 2.17 ± 3.05 | 11.32 ± 7.95 | *W* = 108.50, *p* < 0.001 *** |
| **Ford Insomnia Response to Stress Test (FIRST)** | 15.83 ± 4.19 | 22.91 ± 4.70 | *t* (67) = -6.61, *p* < 0.001 *** |
| **Pre-Sleep Arousal Scale (PSAS)** | 19.57 ± 3.36 | 34.50 ± 8.39 | *W* = 43.00, *p* < 0.001 *** |
| **Trauma Experience Questionnaire (TEQ)** | 0.46 ± 0.83 | 0.42 ± 1.16 | *W* = 551.00, *p* = 0.457 |
| **Reduced Morningness-Eveningness Questionnaire (r-MEQ)** |  |  |  |
| Morningness Type (score > 17) | 8 (11.59%) | 3 (4.35%) |  |
| Eveningness Type (score < 12) | 3 (4.35%) | 12 (17.39%) |  |
| Neither Type (12 ≤ score ≤17) | 24(34.78%) | 19 (27.54%) | $\chi^{2}$(2) = 8.24, *p* = 0.016 * |
| **Written Language** |  |  |  |
| Simplified Chinese | 16 (23.19%) | 15 (21.74%) |  |
| Traditional Chinese | 19 (27.54%) | 19 (27.54%) | $\chi^{2}$(1) = 0.02, *p* = 0.894 |

*Notes.* To compare between group differences, for continuous data, independent sample t-tests (*t*) were conducted if assumption was met. Otherwise, Wilcoxon rank-sum tests (*W*) would be performed instead. For count data, chi-square test ($\chi^{2}$*)* was conducted. **p* < 0.05; ****p* < 0.001.

^a^ Insomnia subtype was obtained from clinical interview.

### Sleep characteristics

**Supplementary Table 3 Sleep characteristics for healthy and insomnia group (N = 69; mean ± SD)**

|  | Heathy  (N = 35) | Insomnia  (N = 34) | Test statistics |
| --- | --- | --- | --- |
| **Sleep Diary^a^** |  |  |  |
| Bedtime | 00:03:56 | 00:11:03 |  |
| Sleep onset | 00:19:21 | 00:36:29 |  |
| Sleep offset | 08:09:34 | 08:14:59 |  |
| Get up time | 08:22:47 | 08:37:13 |  |
| TIB (in min) | 500.22 ± 35.45 | 506.59 ± 60.30 | *W* = 558.50, *p* = 0.666 |
| TST (in min) | 484.98 ± 34.16 | 462.62 ± 52.49 | *t* (67) = 2.10, *p* = 0.039 * |
| SOL (in min) | 11.21 ± 7.05 | 28.75 ± 16.94 | *W* = 140.00, *p* < 0.001 *** |
| WASO (in min) | 4.03 ± 3.95 | 13.74 ± 10.18 | *W* = 206.00, *p* < 0.001 *** |
| SE | 0.97 ± 0.02 | 0.92 ± 0.04 | *W* = 87.00, *p* < 0.001 *** |
| **Sleep Statistics^b^** |  |  |  |
| TIB (in min) | 486.10 ± 11.99 | 486.51 ± 4.47 | *W* = 546.00, *p* = 0.699 |
| TST (in min) | 449.71 ± 28.32 | 430.90 ± 45.76 | *W* = 423.00, *p* = 0.058 |
| SOL (in min) | 16.99 ± 14.99 | 27.03 ± 23.49 | *W* = 401, *p* = 0.030 * |
| WASO (in min) | 19.72 ± 14.40 | 28.88 ± 41.16 | *W* = 534.00, *p* = 0.593 |
| SE | 0.92 ± 0.05 | 0.89 ± 0.09 | *W* = 424.00, *p* = 0.060 |
| N1 (%) | 5.67 ± 2.11 | 5.87 ± 2.98 | *W* = 560.00, *p* = 0.831 |
| N2 (%) | 52.19 ± 8.45 | 54.10 ± 7.50 | *t* (66) = - 0.99, *p* = 0.327 |
| SWS (%) | 16.98 ± 5.94 | 16.00 ± 6.34 | *t* (66) = 0.66, *p* = 0.514 |
| REM (%) | 25.15 ± 7.46 | 24.03 ± 4.93 | *W* = 524.00, *p* = 0.514 |
| Arousal index | 6.45 ± 2.37 | 6.67 ± 2.73 | *W* = 565.00, *p* = 0.879 |
| Arousal Index at N1 | 23.1 ± 10.34 | 20.84 ± 10.95 | t(66) = 0.88, p = 0.384 |
| Arousal Index at N2 | 3.92 ± 1.92 | 3.91 ± 1.85 | W = 573, p = 0.956 |
| Arousal Index at N3 | 1.67 ± 1.87 | 1.35 ± 1.30 | W = 522, p = 0.494 |
| Arousal Index at REM | 6.33 ±3 .27 | 6.9 ± 5.64 | W = 545, p = 0.692 |
| SWS*REM^c^ | 420.73 ± 176.71 | 379.40 ± 150.79 | *t* (66) = 1.04, *p* = 0.303 |

*Notes.* To compare between group differences, for continuous data, independent sample t-tests (*t*) were conducted if assumption was met. Otherwise, Wilcoxon rank-sum tests (*W*) would be performed instead. For count data, chi-square test ($\chi^{2}$*)* was conducted. **p* < 0.05; ****p* < 0.001.
^a^ Sleep diary was used to record the sleep/wake pattern throughout the study period (averaged recording days = 16.9 days); SOL: sleep onset latency, which was defined as the period between “lights out” and the first epoch of sleep stages. WASO: wake after sleep onset; TIB: time in bed; TST: total sleep time; SE: sleep efficiency.

^b^ Sleep statistics were extracted from the sleep score of the second night EEG recording in the laboratory. Data from one healthy participant was corrupted due to technical error, thus the final sample size of sleep EEG for healthy group is 34.

^c^ SWS*REM: the product of %SWS and %REM (Stickgold, 2000; Mednick, 2003).

### Sensitivity Analyses: Participant exclusion based on ±3SD criteria

**Supplementary Table 4 Participant Exclusions Based on ±3 Standard Deviation Criterion**

| **Domains** | **Tasks** | **Outcomes** | **Excluded HC (n)** | **Excluded ID (n)** | **Results after excluding outliers** |
| --- | --- | --- | --- | --- | --- |
| **Memory** | **Mental recall task** | Subjective Recall | 3 | 0 | Results are similar |
|  |  | Objective Recall (include "pass" trial) | 3 | 1 | Group × Emotion insignificant |
|  |  | Objective Recall (exclude "pass" trial) | 3 | 1 | Group × Emotion × Time insignificant |
|  |  | Combining subjective and objective recall | 2 | 1 | Results are similar |
|  | **Verbal recall task** | Identification | 1 | 0 | Results are similar |
|  |  | Gist | 1 | 1 | Group × Time insignificant |
| **Emotion** | **Picture viewing task** | Valence | 1 | 0 | Results are similar |
|  |  | Arousal | 0 | 1 | Results are similar |
|  | **Verbal recall task** | Valence | 0 | 1 | Results are similar |
|  |  | Arousal | 0 | 3 | Results are similar |

In almost all cases, the exclusion affected only a small number of participants (0–3 per group), and the main findings remained stable. In a few tasks (e.g., Objective Recall), exclusion led to slight differences in effect size or significance, though the overall interpretation did not change.

### Model Generalized Variance Inflation Factors (GVIFs)

**Supplementary Table 5 Generalized Variance Inflation Factors**

| **Variable** | **GVIF** | **Df** | **GVIF^(1/(2×Df))** |
| --- | --- | --- | --- |
| Group | 7.56 | 1 | 2.75 |
| Time | 1 | 2 | 1 |
| Emotion | 1 | 1 | 1 |
| Group x Time | 1 | 2 | 1 |
| Group x Emotion | 1 | 1 | 1 |
| Time x Emotion | 1 | 2 | 1 |
| Group x Time x Emotion | 1 | 2 | 1 |
| Sex | 1.24 | 1 | 1.11 |
| scale(Age) | 1.3 | 1 | 1.14 |
| Written language | 1.2 | 1 | 1.1 |
| MEQ_type | 1.48 | 2 | 1.1 |
| scale(BAI) | 2.66 | 1 | 1.63 |
| scale(BDII) | 3.5 | 1 | 1.87 |
| scale(PSAS) | 3.42 | 1 | 1.85 |
| scale(FIRST) | 2.04 | 1 | 1.43 |
| scale(ISI)^a^ | 12.42 | 1 | 3.52 |
| scale(PSQI)^a^ | 5.45 | 1 | 2.34 |

Notes. ^a^ ISI and PSQI were excluded from the full model due to adjusted GIFs greater than 2, strongly correlated with our primary fixed factor Group. MEQ_type: Reduced Morningness–Eveningness Questionnaire (Adan & Almirall, 1991); BAI: Beck Anxiety Inventory (Brown & Epstein, 1988); BDII: Beck Depression Inventory-II (Beck et al., 1996); PSAS: Pre-Sleep Arousal Scale (Nicassio et al., 1985); FIRST: Ford Insomnia Response to Stress Test (Gelaye et al., 2016); Insomnia Severity Index (Morin et al., 2011), Pittsburgh Sleep Quality Index (Buysse et al., 1989).

### Encoding accuracy

Participants were required to learn the pseudo word – picture pairs for at least 4 times to achieve a minimum accuracy of 90% during the 4 testing blocks. One participant from ID group failed to reach the criteria and learned a total of 6 times to achieve at least 80% accuracy. One participant from HC group was identified as a statistical outlier (mean ±3SD) at the lower end. Analyses with and without these two participants did not yield differences, thus the following results were based on the whole dataset (the accuracy from the first 4 blocks were included for the participant learned 6 times).

Linear mixed regression was conducted to the accuracy of each testing block (i.e., sum of correct responses/total responses): Accuracy ~ Group * Emotion * Block + (1|participant).

The full model with covariates was selected. Results showed significant Block and Emotion main effect (*F*(3,469) = 523.00, *p* < 0.001; *F*(1,469) = 12.17, *p* < 0.001), Group*Block (*F*(3,469) = 3.25, *p* = 0.022) and Emotion*Block interaction effect (*F*(3,469) = 4.86, *p* = 0.002). Further inspection on group-related effect suggested that insomnia group performed significantly better than healthy group during the first block of learning (*t*(86.8) = 2.06, *p* = 0.042, see Supplementary Figure 1A-B). The group differences disappeared in the second block and reduced over time (block 2: *t*(86.8) = 1.06, *p*  = 0.294; block 3: *t*(86.8) = 0.70, *p*  = 0.486; block 4: *t*(86.8) = -0.10, *p*  = 0.921). Neutral pictures were overall better encoded than negative pictures (*t*(469) = -3.49, *p* <0.001), mostly during the first block (*t*(469) = -5.01, *p* < 0.001, see Supplementary Figure 1C-D). This was consistent with previous study suggesting that emotional stimuli might weaken context dependent memory, resulting in impaired associations between negative items and their neutral cues (Bisby & Burgess, 2014).

Among the covariates, MEQ type and Gender was significant (*F*(2,58) = 5.17, *p* = 0.009; *F*(1,58) = 4.83, *p* = 0.032). Further inspection suggested that morningness individual performed much worse than individual with neither type (*t*(58) = -3.21, *p*  = 0.002). No differences were found between morning and evening type (*t*(58) = -1.99, *p*  = 0.051) or between evening and neither type (*t*(58) = -0.71, *p*  = 0.482). This result suggested morningness-eveningness type affects the ability of learning happened in the evening. In addition, female showed higher encoding accuracy than male (*t*(58) = 2.20, *p*  = 0.032).

*Notes.* Encoding accuracy across groups. **A/C.** Violin plot showed the density of the data distribution at participant level. The box plot showed the mean (middle bold line) and 1 standard error (upper and lower boundary). **B/D**. Interaction effects extracted from the linear mixed regression at trial level. **p* < 0.05, ****p* < 0.001

**Supplementary Figure 1.** Encoding behavioral results

### Memory performance

**
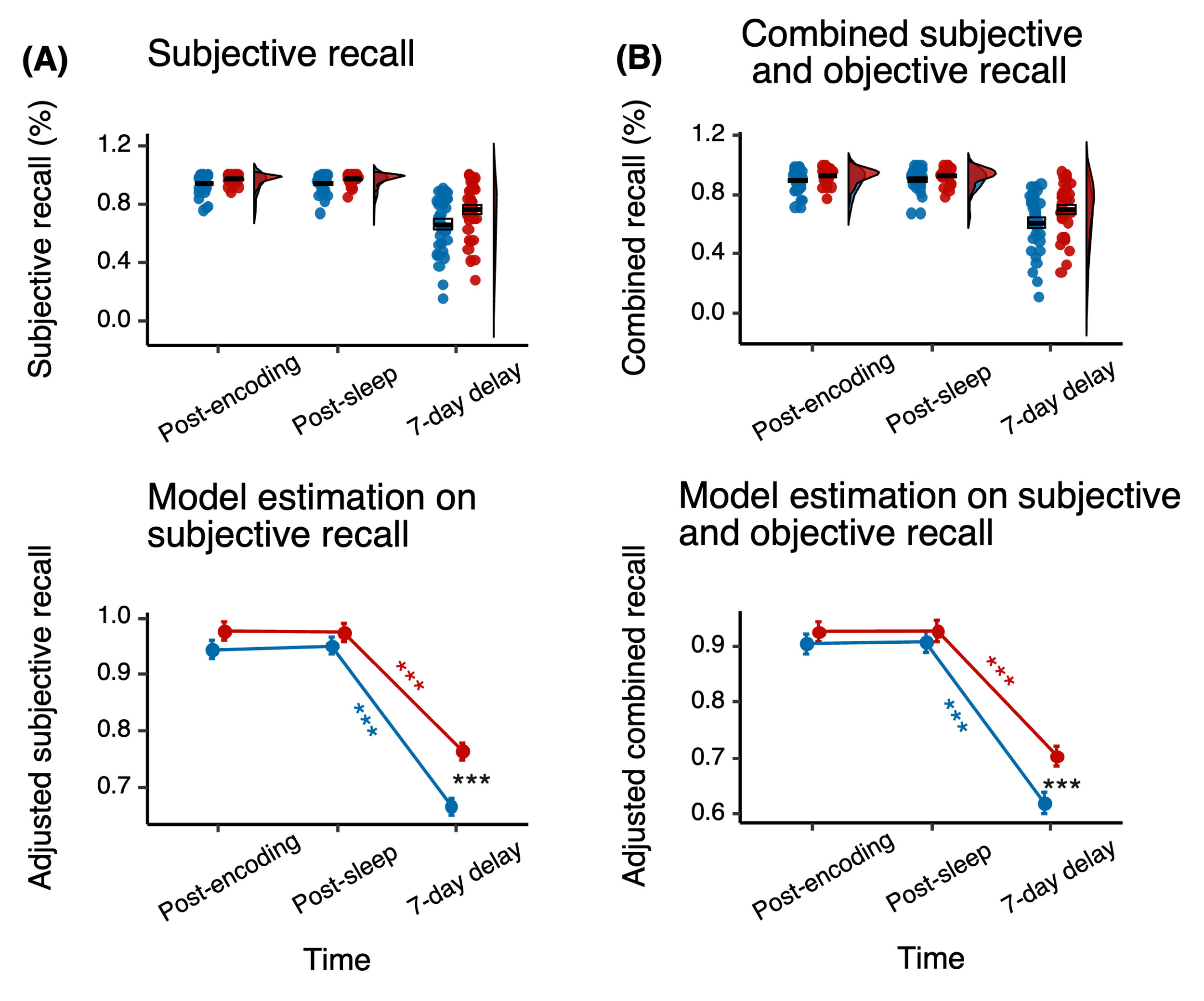
Supplementary Figure 2.** Memory performance of subjective recall scores and combined subjective and objective recall scores.

*Notes.* The Group*Time effect on subjective recall and combined recall scores. Across multiple memory indices, compared to insomnia group, healthy group showed much more memory decay from post-sleep to 7-day delay, resulting in a significant group difference at 7-day delay session. Upper panel shows violin plot showed the density of the data distribution at participant level. The box plot showed the mean (middle bold line) and 1 standard error (upper and lower boundary). Lower panel shows interaction effects extracted from the regression at trial level. **p* < 0.05, ***p* < 0.01, ****p* < 0.001

**Supplementary Figure 3.** Group $\boldsymbol{\times}$ Emotion effect on Objective Recall (include “pass” trials)

*Notes.* For negative memories, participants with ID exhibited a better memory performance than HC, while no group differences were found for neutral memories. Upper panel shows violin plot showed the density of the data distribution at participant level. The box plot showed the mean (middle bold line) and 1 standard error (upper and lower boundary). Lower panel shows interaction effects extracted from the regression at trial level. **p* < 0.05, ***p* < 0.01, ****p* < 0.001

#### Correlation between scores from mental recall and verbal recall tasks

To examine consistency between the subjective report from **mental recall task** (EEG responses as primary outcomes) and the oral description from **verbal recall task**, we additionally ran Spearman correlation analyses on these measurements. The results demonstrated strong and statistically significant correlations between objective recall judgments from the mental recall task and both identity and gist scores from the verbal recall task across all sessions and groups (*ρ* = 0.44–0.81, all *p* < 0.01; see Supplementary Table 6). These findings provide compelling evidence for the reliability and validity of participants’ objective recall responses during the EEG mental recall tasks.

Subjective recall judgments also showed moderate-to-strong correlations, particularly at the 7-day delayed session (*ρ* = 0.70–0.81, all *p* < 0.001), suggesting that participants' sense of successful memory retrieval was largely aligned with independently verified recall quality. However, weaker correlations were observed during the post-sleep session, especially between subjective recall and gist scores in healthy controls (*ρ* = 0.12, *p* = 0.516), indicating some variability in early-stage recall confidence.

These analyses support the overall **convergent validity** of our mental recall task measures—especially the objective recall judgments—and reinforce our interpretation of EEG-based memory representations on these correct objective recall trials as reflecting meaningful retrieval processes.

| **Supplementary Table 6** **Correlation between scores from Recall and Verbal Recall tasks** | | | | | | |
| --- | --- | --- | --- | --- | --- | --- |
| **Sessions** | **Group** | **n** | **Recall measures** | **Verbal recall measures** | **Spearman_rho** | **p value** |
| Post-sleep | HC | 34 | Objective Recall | Identification Scores | 0.45 | 0.008 |
| Post-sleep | ID | 34 | Objective Recall | Identification Scores | 0.67 | <0.001 |
| Post-sleep | HC | 34 | Objective Recall | Gist Scores | 0.44 | 0.01 |
| Post-sleep | ID | 34 | Objective Recall | Gist Scores | 0.55 | <0.001 |
| 7-day delayed | HC | 35 | Objective Recall | Identification Scores | 0.81 | <0.001 |
| 7-day delayed | ID | 34 | Objective Recall | Identification Scores | 0.76 | <0.001 |
| 7-day delayed | HC | 35 | Objective Recall | Gist Scores | 0.74 | <0.001 |
| 7-day delayed | ID | 34 | Objective Recall | Gist Scores | 0.74 | <0.001 |
| Post-sleep | HC | 34 | Subjective Recall | Identification Scores | 0.34 | 0.047 |
| Post-sleep | ID | 34 | Subjective Recall | Identification Scores | 0.55 | <0.001 |
| Post-sleep | HC | 34 | Subjective Recall | Gist Scores | 0.12 | 0.516 |
| Post-sleep | ID | 34 | Subjective Recall | Gist Scores | 0.22 | 0.205 |
| 7-day delayed | HC | 35 | Subjective Recall | Identification Scores | 0.81 | <0.001 |
| 7-day delayed | ID | 34 | Subjective Recall | Identification Scores | 0.71 | <0.001 |
| 7-day delayed | HC | 35 | Subjective Recall | Gist Scores | 0.7 | <0.001 |
| 7-day delayed | ID | 34 | Subjective Recall | Gist Scores | 0.71 | <0.001 |

### Correlation analyses on sleep and memory decay

**Supplementary Figure 4.** Correlation between SWS_REM product and negative objective recall scores (exclude pass trials).

*Notes.* Spearman correlation results with FDR corrected *p* values are presented in the figure. Excluding ±3SD outliers (i.e., the two empty dots), the correlation for healthy group became significant (*r_s_* = -0.47, FDR corrected *p* = 0.045).

### Regression analyses on sleep and memory decay

To further investigate the unique role of SWS*REM in memory decay, while controlling for SWS and REM individually, we performed backward stepwise model selection on a robust regression: memory decay ~ SWS + REM + SWS*REM. The full model was the optimal model with the least Robust Final Prediction Error (RFPE). SWS*REM was a significant predictor ($b$= 0.001, *p* = 0.035).

### Baseline valence/arousal ratings from the picture rating task

#### Valence ratings

The full model with covariates was selected. The Group*Emotion effect was not significant ($\chi^{2}(1) = 3.61$, *p* = 0.058, see Supplementary Figure 5A-B). Interestingly, age as a covariate significantly predicted the valence ratings ($\beta$ = -0.23, *p* = 0.002), suggesting individuals with older age gave lower valence ratings (more negative) in general.

One potential outlier from healthy group was identified (±3SD) and excluding this participant at trial-level analysis did not influence the results above.

#### Arousal ratings

The basic model was selected. A significant Group*Emotion effect was found ($\chi^{2}$(1) = 35.78, *p* < 0.001, see Supplementary Figure 5C-D). The Post-hoc analysis exhibited that insomnia patients rated negative pictures with higher arousal than healthy controls (*z* = -2.43, *p* = 0.015) while no group differences were found in rating neutral pictures (*z* = -0.99, *p* = 0.323).

One insomnia participant was identified as a potential outlier at the participant level. Excluding this participant from trial-level analysis did not change the results.

#### Neural representation during initial picture viewing

We next conducted multivariate ERP-based decoding analyses to delineate the neuro-temporal processing when participants viewed negative vs. neutral pictures. Results suggested that while HC showed a stable neural representation that distinguished between negative and neutral pictures between 250 – 2000 ms (1 cluster, *p* = 0.004, corrected from 10,000 permutations, Figure 4A), ID showed a relatively earlier yet shorter neural representation of emotion during 190 – 1260ms (1 cluster, corrected *p* = 0.026, Supplementary Figure 6). However, between-group permutation did not reveal any significant group differences (see Supplementary Figure 6).

Overall insomnia patients perceived negative pictures with higher arousal than healthy controls, whereas no significant group differences were found in valence ratings.

*Notes.* Valence/arousal rating at baseline across groups. **A/C.** Violin plot showed the density of the data distribution at participant level. The box plot showed the mean (middle bold line) and 1 standard error (upper and lower boundary). **B/D.** Interaction effects extracted from the ordinal logistic regression at trial level. **p* < 0.05, ****p* < 0.001

**Supplementary Figure 5** Picture rating behavioral results

### Correlation analyses on sleep and emotion dissipation

**Supplementary Figure 7.** Correlation between REM sleep (A) SWS_REM product (B) and negative arousal ratings.
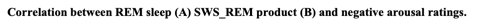
*.*

*Notes.* To investigate valence-specific neural representations during the picture rating task, we performed ERP-based multivariate pattern classification (decoding) to distinguish between emotions (negative vs. neutral pictures) in healthy and insomnia groups. The chance level was set at 0.5. A significant cluster indicates that emotional information could be successfully decoded from participants’ whole-brain EEG patterns. **A-B.** Multivariate decoding on negative vs. neutral pictures across temporal in healthy and insomnia group respectively. **C.** Between-group t-value on the decoding accuracy across temporal (adjusted by non-parametric permutation). Bounded areas represented decoding accuracy significantly larger than 0.5, *p* < 0.05 (two-tailed).

**Supplementary Figure 6** Multivariate pattern classification for picture rating task*.*

**(A)**

**(B)**

**(C)**

*Notes.* Spearman correlation results with FDR corrected *p* values are presented in the figure. Excluding ±3SD outliers (i.e., the empty dots), the significance remains unchanged (FDR corrected *p*s = 0.048)

### Affective ratings accounting for memory scores during post-sleep and 7-days delayed verbal recall tasks

#### Valence ratings

The basic model was the optimal model. We found significant Emotion, Time, Identification main effect ($\chi^{2}$(1) = 92.08, *p* < 0.001; $\chi^{2}$(1) = 29.71, *p* < 0.001; $\chi^{2}$(1) = 8.30, *p* = 0.004), Time*Emotion ($\chi^{2}$(1) = 61.96, *p* < 0.001), Emotion*Identification ($\chi^{2}$(1) = 216.31, *p* < 0.001), Time*Identification ($\chi^{2}$(1) = 12.90, *p* < 0.001), Emotion*Time*Identification ($\chi^{2}$(1) = 25.82, *p* < 0.001) and Group*Emotion*Identification interaction effects ($\chi^{2}$(1) = 8.66, *p* = 0.003, Supplementary Figure 8A-B).

To decompose the significant Group*Emotion*Identification interaction, we examined Group*Emotion on remembered (identification = 1) and forgotten (identification = 0) trials separately. For remembered trials, Group*Emotion was significant ($\chi^{2}$(1) = 15.46, *p* < 0.001), suggesting that the difference between negative and neutral memory valence ratings given by healthy group was greater than those given by insomnia group (ID: *z* = -16.42, *p* < 0.001; HC: *z* = -18.40, *p* < 0.001). No group differences were found in neither negative nor neutral memories (*p* > 0.173). For forgotten trials, the Group*Emotion was not significant ($\chi^{2}$(1) = 0.60, *p* = 0.440).

In addition to group-related effects, the Emotion and Identification main effect suggested that in general the individuals rated negative/remembered memories more negatively than neutral/forgotten memories (negative vs. neutral: *z* = -13.15, *p* < 0.001; remembered vs. forgotten: *z* = -2.89, *p* = 0.004). Time main effect suggested that participants gave more positive valence ratings from post-sleep to 7-day delayed session (*z* = -5.44, *p* < 0.001), which was further quantified by Time*Emotion effect, such that the over-time increase on valence ratings were specifically observed in negative memories (*z* = -11.22, *p* < 0.001), but not in neutral memories (*z* = 1.48, *p* = 0.139). Time*Identification effect suggested that the over-time increased valence ratings were mostly driven by remembered trials (*z* = -4.74, *p* < 0.001) and to a less extent for forgotten trials (*z* = -2.98, *p* = 0.003), which led to a significant remembered vs. forgotten difference during the 7-day delayed session (*z* = -6.68, *p* < 0.001) but not the post-sleep session (*z* = 0.34, *p* = 0.731). The Identification effect was also modulated by Emotion (i.e., Emotion*Identification), such that remembered negative memories were rated more negatively than forgotten negative memories (*z* = -14.53, *p* < 0.001). For neutral memories, remembered neutral trials were rated more positively than forgotten trials (*z* = 6.98, *p* < 0.001). Together these led to a larger negative vs. neutral distinction for remembered trials (*z* = -20.96, *p* < 0.001) than for forgotten trials (*z* = -4.83, *p* < 0.001), suggesting the emotional valence was reduced if people forget the content.

The abovementioned two-way interactions was further quantified by the Emotion*Time*Identification effect: the over-time valence increases were observed only in negative remembered and forgotten trials (*z* = -5.95, *p* < 0.001; *z* = -9.82, *p* < 0.001) but not in neutral remembered and forgotten trials (*z* = 0.97, *p* = 0.334; *z* = 1.25, *p* = 0.212). This led to different valence rating effects across memory and time: for remembered trials, the negative < neutral valence differences were observed in both post-sleep (*z* = -21.65, *p*  < 0.001) and 7-day delayed sessions (*z* = -18.45, *p*  < 0.001); while for forgotten trials, this distinction was only observed in post-sleep session (*z* = -6.56, *p*  < 0.001) but not the 7-day delayed session (*z* = 0.20, *p*  = 0.843).

Two insomnia participants were identified as potential outliers, but sensitivity analysis showed that excluding them from the trial-level analysis did not influence the results.

#### Arousal ratings

The parsimonious model was selected. We observed significant main effects of Emotion and Time ($\chi^{2}$(1) = 71.87, *p* < 0.001; $\chi^{2}$(1) = 6.46, *p* = 0.011), as well as Group*Identification ($\chi^{2}$(1) = 5.39, *p* = 0.021), Emotion*Time ($\chi^{2}$(1) = 37.34, *p* < 0.001), Emotion*Identification ($\chi^{2}$(1) = 146.85, *p* < 0.001), Time*Identification ($\chi^{2}$(1) = 15.51, *p* < 0.001), Emotion*Time*Identification interaction effects ($\chi^{2}$(1) = 10.76, *p* = 0.001). Further inspection of the Group * Identification interaction effect suggested that healthy group tended to rate those remembered trials more arousing than those forgotten (*z* = 1.47, *p*  = 0.141) while the insomnia group showed an opposite pattern: they tended to give more arousing ratings to those forgotten trials than remembered trials (*z* = 1.76, *p*  = 0.079). Note that these did not reach statistical significance. No significant group differences were found (*p* > 0.389).

Moreover, to be comparable with the results from valence ratings, we also took a further look at the non-significant Group*Emotion*Identification ($\chi^{2}$(1) = 2.89, *p* = 0.089, see Supplementary Figure 8C-D). We examined Group*Emotion on remembered (identification = 1) and forgotten (identification = 0) trials separately. For remembered trials, Group*Emotion was significant ($\chi^{2}$(1) = 23.89, *p* < 0.001) and the patten was very similar to the valence results: compared to healthy group, insomnia group gave a less distinct arousal ratings to negative and neutral memories (HC: *z* = 17.14, *p* < 0.001; ID: *z* = 13.38, *p* < 0.001). This was presumably driven by a lower arousal rating to negative memories given by the insomnia group than healthy group (*z* = -1.84, *p* = 0.065, neutral: *z* = 0.31, *p* = 0.759). For forgotten trials, the Group*Emotion was not significant ($\chi^{2}$(1) = 0.13, *p* = 0.716).

In addition to group-related effects, the Emotion main effect suggested participants gave higher arousal ratings to negative than neutral memories (*z* = 10.88, *p*  < 0.001). The Time main effect suggested that participants' arousal ratings significantly reduced from post-sleep to 7-day delayed session (*z* = 2.54, *p* = 0.011), which was modulated by Emotion (i.e., Time*Emotion) suggesting that arousal ratings for negative memories reduced significantly from post-sleep to 7-day delayed session (*z* = 6.47, *p* < 0.001), while for neutral memories, the arousal ratings increased over time (*z* = -2.40, *p* = 0.016). Time*Identification effect suggested that the over-time arousal reduction was particularly observed in forgotten trials (*z* = 3.43, *p* < 0.001), while for remembered trials, the arousal ratings increased over time (*z* = -2.16, *p* = 0.031). This was driven by the Identification difference at different sessions: during the post-sleep session, remembered trials were rated less arousing than forgotten trials (*z* = -2.43, *p* = 0.015), while in the 7-day delayed session, the pattern was opposite so that forgotten trials were rated less arousing than remembered trials (*z* = -3.72, *p* < 0.001). Emotion effect was also modulated by Identification (i.e., Emotion*Identification): participants gave more distinct arousal ratings between negative and neutral remembered trials (*z* = 19.62, *p*  < 0.001) than those forgotten trials (*z* = 2.68, *p*  = 0.008). This was due to negative forgotten trials were rated less arousal than negative remembered trials (*z* = -8.67, *p*  < 0.001) and conversely, neutral forgotten trials were rated more arousal than neutral remember trials (*z* = 8.30, *p*  < 0.001). In other words, forgotten negative and neutral trials were rated more closely to each other. This potentially implicated that when people forget memories, the associated emotion still exists but fades away compared to remembered ones.

The above mentioned two-way interactions could be further quantified by the Time*Emotion*Identification effect: the over-time arousal reduction observed in negative memories was specifically driven by forgotten trials (*z* = 6.31, *p* < 0.001) but not remembered trials (*z* = 1.55, *p* = 0.120). On the contrary, the over-time arousal increase observed in neutral memories was only contributed by remembered trials (*z* = -4.55, *p* < 0.001) but not forgotten trials (*z* = -1.02, *p* = 0.307). These over-time changes led to the emotion differences observed in different sessions: for remembered trials, the distinctive arousal ratings between negative and neutral memories (i.e., negative > neutral) persisted in both post-sleep (*z* = 20.21, *p*  < 0.001) and 7-day delayed sessions (*z* = 16.10, *p*  < 0.001). For forgotten trials, this distinction was only observed during post-sleep (*z* = 4.20, *p*  < 0.001) but not 7-day delayed session (*z* = -1.59, *p*  = 0.113). Collaborating with the Time*Emotion*Identification effect from valence ratings, these results potentially suggested that the emotional valence and arousal were influenced by time and whether individuals remembered the content: 7 days after encoding, we observed the emotion tones dissipated for forgotten memories.

One healthy and one insomnia participants were identified as outliers at participant-level. Trial-level analysis excluding these two participants did not yield any differences from the above results.

Taken together, when controlling participants’ memory performance, we observed group differences when rating remembered pictures: compared to healthy group, insomnia group tended to give a less distinct valence and arousal ratings to negative vs. neutral memories. Caveats shall be noted for the three-way interaction effect in arousal ratings, which did not reach statistical significance (*p* = 0.089).

*Notes.* Valence/Arousal rating at verbal recall across emotion and identification. **A/C.** Violin plot showed the density of the data distribution at participant level. The box plot showed the mean (middle bold line) and 1 standard error (upper and lower boundary). **B/D.** Interaction effects extracted from the ordinal logistic regression at trial level. **p* < 0.05, ***p* < 0.01, ****p* < 0.001

**Supplementary Figure 8** Verbal recall emotion behavioral results accounting for memory scores

### ERP results

ERPs implicating in emotional processing was extracted during the picture viewing tasks and recall tasks (i.e., P300 and Late Positive Components [LPP]). Specifically, we extracted the mean amplitudes of 300-800ms (i.e., early) and 1000-2000ms (i.e., late component) across electrodes over frontal central (i.e., FCz, FC1/2, FC3/4, Cz, C1/2, C3/4) and parietal central (i.e., CP1/2, CP3/4, Pz, P1/2, P3/4) regions (Dolcos & Cabeza, 2002; Hajcak, Macnamara, & Olvet, 2010; Hajcak, Weinberg, MacNamara, & Foti, 2012).

For statistical testing of the ERPs from picture rating task, we conducted 2 (Group: healthy vs. insomnia group) × 2 (Emotion: negative vs. neutral pictures) mixed ANOVA on ERP amplitudes.

For the ERPs from the recall tasks, we are interested in the emotional representation based on the remembered trials. Thus, we only included correct trials based on the objective recall (i.e., trials participants correctly identified the emotional content of the associated picture), and on the combination of subjective and objective recall (i.e., participants indicated that they remembered and correctly identified the emotional content of the associated picture). For statistical testing, we conducted 2 (Group: healthy vs. insomnia group) × 2 (Emotion: negative vs. neutral pictures) mixed ANOVA on ERP amplitudes at different sessions.

**Supplementary Table 7 Participant exclusions based on >20% EEG trials affected by artifacts**

| **Tasks** | **Excluded HC (n)** | **Excluded ID (n)** | **HC (n)** | **ID (n)** |
| --- | --- | --- | --- | --- |
| Picture rating task | 1 | 1 | 34 | 33 |
| Mental recall #1 | 1 | 1 | 34 | 33 |
| Mental recall #2 | 0 | 1 | 35 | 33 |
| Mental recall #3 | 3 | 0 | 32 | 33 |

Note. The total number of ID is 34. The EEG of one ID participants from mental recall #3 had missing triggers thus being excluded from analysis

#### P300 and LPP during the picture rating task

Results found a significant Group*Emotion effect for P300 at the frontal central area (i.e., FCz, FC1/2, FC3/4, Cz, C1/2, C3/4; *F*(1,65) = 8.55, *p* = 0.005, see Supplementary Figure 9A). Specifically, healthy group exhibited significant higher P300 amplitudes to negative than neutral pictures (*t*(65) = 2.50, *p* = 0.015), while insomnia group showed comparable amplitudes to both negative and neutral pictures (*t*(65) = -1.65, *p* = 0.104). Such pattern continued into the late component (i.e., 1000-2000ms) at frontal central regions but the overall interaction effect did not reach statistical significance (*F*(1,65) = 2.79, *p* = 0.100).

ANOVAs on the P300 and LPP at parietal central areas did not reveal any group-related effect (*p* > 0.328). The main effect of Emotion was significant (early: *F*(1,65) = 50.62, *p* < 0.001; late: *F* (1,65) = 58.37, *p* < 0.001), suggesting both groups exhibited a higher amplitude when viewing negative compared to neutral pictures at parietal central regions.

Collaborating the visual inspection of the topography for the Emotion effect at 300-800ms and 1000-2000ms (see Supplementary Figure 9C), while healthy group exhibited emotion-related ERPs at both frontal central and parietal central regions, this emotion effect was dominantly found at parietal regions in insomnia group.


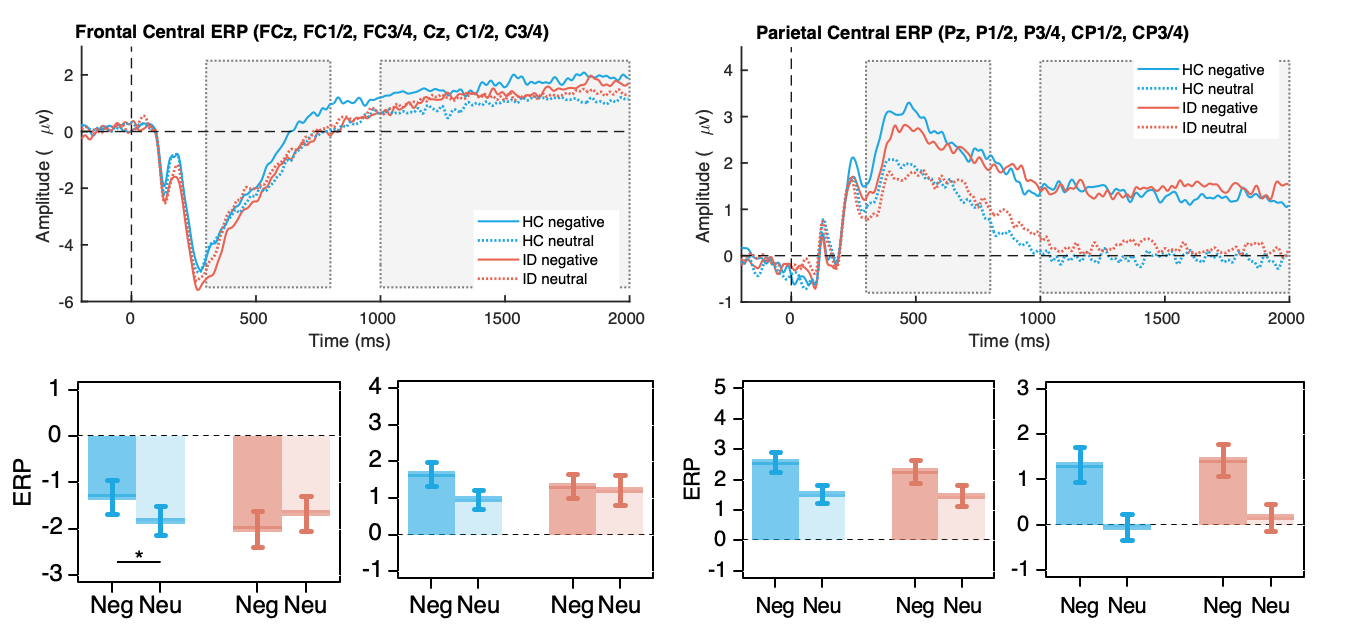


**A**

**B**

**C**

**ERP topography**

**Supplementary Figure 9** Picture rating ERP results

*Notes*. ERP results extracted from picture rating task. **A-B.** Upper panel is the ERP waves at frontal central and parietal central electrodes respectively. The dotted boxes are the time window of interest: early P300: 300-800ms and LPP: 1000-2000ms. Lower panel is the barplots visualizing the mean amplitudes of the interested components. **p <* 0.05. **C.** The topography for emotion effects (i.e., negative – neutral) of the interested components for healthy and insomnia respectively.

#### P300 and LPP during the mental recall tasks

We extracted trials based on correct objective recall (see Supplementary Figure 10) and combined subjective and objective recall (see Supplementary Figure 11).

*Correct objective recall.*  The Group*Emotion effect was significant at the central parietal regions from 1000-2000ms during the post-encoding recall (*F* (1,65) = 4.15, *p* = 0.046, see Supplementary Figure 10B). Pairwise comparisons did not reveal any significance, yet the numerical pattern for emotion effect in insomnia and healthy group was opposite: while healthy group elicited higher amplitude for negative memory cues than neutral ones (*t*(65) = 1.19, *p =* 0.238), insomnia group showed higher amplitude for neutral than negative ones (*t*(65) = -1.69, *p =* 0.097). No other group-related effects were significant (*p*s > 0.065).

*Combined subjective and objective recall.*  The Emotion main effect was found to be significant at 1000-2000ms at frontal central regions during the post-encoding session (*F* (1,65) = 4.68, *p* = 0.034, see Supplementary Figure 11A), suggesting both groups exhibited higher LPP amplitudes to neutral memory cue words than negative ones. The Group*Emotion effect at the central parietal regions from 1000-2000ms during the post-encoding recall was not significant (*F* (1,65) = 3.63, *p* = 0.061). However, the pattern was in accord with the results from correct objective recall trials (see Supplementary Figure 11B barplot). No other group-related effects were significant (*ps* > 0.098).

Collectively, the univariate ERPs from recall tasks revealed limited between-group differences. Therefore, we conducted multivariate pattern classifications on these ERPs using whole-brain electrodes, aiming to reveal emotion representational patterns in both groups and how they transform over time (see main text and the following section).

**Supplementary Figure 10** Recall ERP waveforms based on correct objective recall trials

*Notes.* ERP waveforms extracted from the three recall tasks that occurred during post-encoding (recall #1), post-sleep (recall #2) and 7-day delayed sessions (recall #3). Averaged ERPs were based on trials that were correctly responded for objective recall questions, that is, participants correctly identified whether the picture had negative scenes/events involved. **A-B.** The ERP waves at frontal central and parietal central electrodes respectively. The cue words were presented from 0 – 1000ms. The dotted boxes are the time window of interest: early P300: 300-800ms and LPP: 1000-2000ms.

**A**

**B**

Neg Neu

Neg Neu

*Notes.* ERP waveforms extracted from the three recall tasks that occurred during post-encoding (recall #1), post-sleep (recall #2) and 7-day delayed sessions (recall #3). Averaged ERPs were based on trials that were correctly responded for both subjective and objective recall questions, that is, participants indicated that they successfully recalled the associated pictures and correctly identified whether the picture had negative scenes/events involved. **A-B.** The ERP waves at frontal central and parietal central electrodes respectively. The cue words were presented from 0 – 1000ms. The dotted boxes are the time window of interest: early P300: 300-800ms and LPP: 1000-2000ms.

**Supplementary Figure 11** Recall ERP waveforms based on correct subjective and objective recall trials

**A**

**B**

Neg Neu

Neg Neu

### ERP-based decoding results for recall tasks based on correct subjective and objective recall trials

Consistent with decoding results based on correct objective recall trials, only insomnia group showed significant decoding clusters (see Supplementary Figure 12). For same-session decoding, insomnia group showed a significant cluster during post-encoding recall from around 440 to 3000 ms (1 cluster, *p* = 0.016). Between-group comparison also suggested a significant group difference (1 cluster see Supplementary Figure 12B, *p* = 0.047).

For between-session decoding, similar to results based on correct objective recall trials, there are significant clusters when using post-sleep recall data at around 860-3000 ms to decode 7-day delayed recall at around 120 – 1660ms (1 cluster, *p* = 0.017) and vice versa (i.e., data from 7-day delayed recall at around -120 – 1640 ms can significantly decode data from post-sleep recall data at around 500 – 3000 ms).


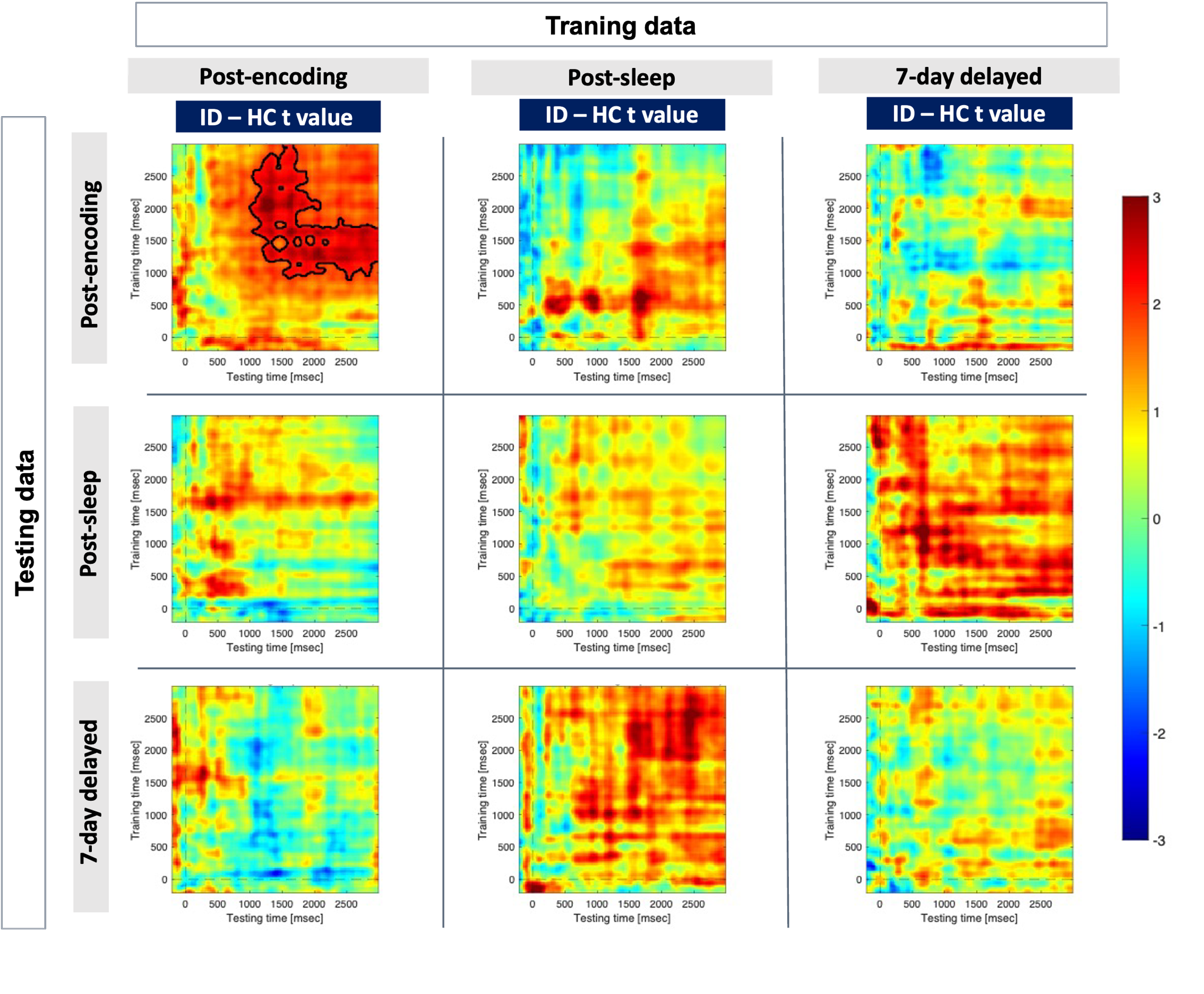


**Supplementary Figure 12** Multivariate pattern classification for recall tasks based on correct subjective and objective recall trials

**A Multivariate decoding on the mental recall tasks within insomnia and healthy groups respectively**

**B Group-level differences in decoding accuracy (ID minus HC) for each time–time matrix**

*Notes*. **A**. Multivariate decoding on negative vs. neutral memory cues across temporal in insomnia and healthy group respectively. Bounded areas represented decoding accuracy significantly larger than 0.5, *p* < 0.05 (two-tailed). **B.** Between-group t-value on the decoding accuracy across temporal (adjusted by non-parametric permutation) and across sessions. Bounded areas represented decoding accuracy significantly differ between groups (ID-HC), *p* < 0.05 (two-tailed). HC: Healthy Controls; ID: Insomnia Disorder

## References

Adan, A., & Almirall, H. (1991). Horne & Östberg morningness-eveningness questionnaire: A reduced scale. *Personality and Individual Differences*, *12*(3), 241–253. doi: 10.1016/0191-8869(91)90110-W

American Academy of Sleep Medicine. (2005). The international classification of sleep disorders. *Diagnostic and Coding Manual*, 148–152.

American Psychiatric Association. (2013). Diagnostic and Statistical Manual of Mental Disorders, 5th Edition. In *Diagnostic and Statistical Manual of Mental Disorders, 5th Edition*. doi: 10.1176/appi.books.9780890425596.744053

Beck, A. T., Steer, R. A., & Brown, G. K. (1996). Manual for the Beck depression inventory-II. *San Antonio, TX: Psychological Corporation*.

Bisby, J. A., & Burgess, N. (2014). Negative affect impairs associative memory but not item memory. *Learning and Memory*, *21*(1), 21–27. doi: 10.1101/lm.032409.113

Brown, G., & Epstein, N. (1988). An Inventory for Measuring Clinical Anxiety: Psychometric Properties. *Journal of Consulting and Clinical Psychology*, *56*(6), 893–897.

Buysse, D. J., Reynolds, C. F., Monk, T. H., Berman, S. R., & Kupfer, D. J. (1989). The Pittsburgh sleep quality index: A new instrument for psychiatric practice and research. *Psychiatry Research*. doi: 10.1016/0165-1781(89)90047-4

Christensen, R. H. B. (2022). *ordinal—Regression Models for Ordinal Data*. R package version 2022.11-16.

Dolcos, F., & Cabeza, R. R. (2002). Event-related potentials of emotional memory: Encoding pleasant, unpleasant, and neutral pictures. *Cognitive, Affective, & Behavioral Neuroscience*, *2*(3), 252–263. doi: 10.3758/CABN.2.3.252

Gelaye, B., Zhong, Q. Y., Barrios, Y. V., Redline, S., Drake, C. L., & Williams, M. A. (2016). Psychometric evaluation of the ford insomnia response to stress test (FIRST) in early pregnancy. *Journal of Clinical Sleep Medicine*, *12*(4), 579–587. doi: 10.5664/jcsm.5696

Hajcak, G., Macnamara, A., & Olvet, D. M. (2010). Event-related potentials, emotion, and emotion regulation: An integrative review. *Developmental Neuropsychology*, *35*(2), 129–155. doi: 10.1080/87565640903526504

Hajcak, G., Weinberg, A., MacNamara, A., & Foti, D. (2012). ERPs and the Study of Emotion. In *The Oxford Handbook of Event-Related Potential Components*. doi: 10.1093/oxfordhb/9780195374148.013.0222

Küpper, C. S., Benoit, R. G., Dalgleish, T., & Anderson, M. C. (2014). Direct suppression as a mechanism for controlling unpleasant memories in daily life. *Journal of Experimental Psychology: General*, *143*(4), 1443–1449. doi: 10.1037/a0036518

Lang, P. J., Bradley, M. M., & Cuthbert, B. N. (2008). International affective picture system (IAPS): Affective ratings of pictures and instruction manual. *Technical Report A-8, University of Florida, Gainesville, FL*.

Marchewka, A., Żurawski, Ł., Jednoróg, K., & Grabowska, A. (2014). The Nencki Affective Picture System (NAPS): Introduction to a novel, standardized, wide-range, high-quality, realistic picture database. *Behavior Research Methods*, *46*(2), 596–610. doi: 10.3758/s13428-013-0379-1

Merikangas, K. R., Zhang, J., Emsellem, H., Swanson, S. A., Vgontzas, A., Belouad, F., … Mignot, E. (2014). The structured Diagnostic Interview for Sleep Patterns and Disorders: Rationale and initial evaluation. *Sleep Medicine*, *15*(5), 530–535. doi: 10.1016/j.sleep.2013.10.011

Morin, C. M., Belleville, G., Bélanger, L., & Ivers, H. (2011). The insomnia severity index: Psychometric indicators to detect insomnia cases and evaluate treatment response. *Sleep*, *34*(5), 601–608. doi: 10.1093/sleep/34.5.601

Nicassio, P. M., Mendlowitz, D. R., Fussell, J. J., & Petras, L. (1985). The phenomenology of the pre-sleep state: The development of the pre-sleep arousal scale. *Behaviour Research and Therapy*, *23*(3), 263–271. doi: 10.1016/0005-7967(85)90004-X

Oostenveld, R., Fries, P., Maris, E., & Schoffelen, J.-M. (2011). FieldTrip: Open Source Software for Advanced Analysis of MEG, EEG, and Invasive Electrophysiological Data. *Computational Intelligence and Neuroscience*, *2011*. doi: 10.1155/2011/156869

Sheehan, D. V, Lecrubier, Y., Sheehan, K. H., Amorim, P., Janavs, J., Weiller, E., … Dunbar, G. C. (1998). The Mini-International Neuropsychiatric Interview (M.I.N.I.): The development and validation of a structured diagnostic psychiatric interview for DSM-IV and ICD-10. *The Journal of Clinical Psychiatry*, *59 Suppl 2*, 22–57.
